# Correction: The center cannot hold: A Bayesian chronology for the collapse of Tiwanaku

**DOI:** 10.1371/journal.pone.0305498

**Published:** 2024-06-11

**Authors:** The PLOS ONE Staff

## Notice of republication

An incorrect version of the Supporting Information and Figure files were published in error. The publisher apologizes for this error. This article was republished on January 18, 2024, to correct for this error. Please download this article again to view the correct version.
