# Supplementary material for: The center cannot hold: A Bayesian chronology for the collapse of Tiwanaku
Source: PLoS One. 2023 Nov 22;18(11):e0288798. doi: 10.1371/journal.pone.0288798 (PMC10664893; doi:10.1371/journal.pone.0288798)
Supplement: S1 File — (PDF) [file pone.0288798.s002.pdf]

Supplementary file for:

Marsh EJ, Vranich A, Blom D, Bruno M, Davis K, Augustine J, et al. The center cannot hold: A Bayesian chronology for the collapse of Tiwanaku. PLOS ONE. 2023;18: e0288798. doi:10.1371/journal.pone.0288798

S1. Materials and methods details

Site-specific mixed calibration curve

Radiocarbon calibration curves are hemisphere-specific, but over parts of South America, the summer monsoon mixes air from both hemispheres. In many cases, this mixture is unknown, so the best practice is to use a uniform mixed curve that allows for the full range of air mixtures [1]. Recently, Ancapichun et al. [2, 3] have developed a method for quantifying the relative contributions of air parcels from each hemisphere at specific locations, based on backward trajectory analysis with a HYSPLIT (HYbrid Single-Particle Lagrangian Integrated Trajectory) climate model [4] (Figs S2.1–3). We use the results from October–March, which includes the growing season, when plants take up more ^{14}C from the atmosphere. Shifting this period to exclude November or February has no significant impact on the results (Table S2.1). Finally, we used the $\delta^{18}O$ record from the Quelccaya ice cap [5] to evaluate whether the climate was broadly similar during the Tiwanaku period. A z-test shows no significant difference between the modern period and the running average for AD 300–1150, which covers the period discussed in the paper.

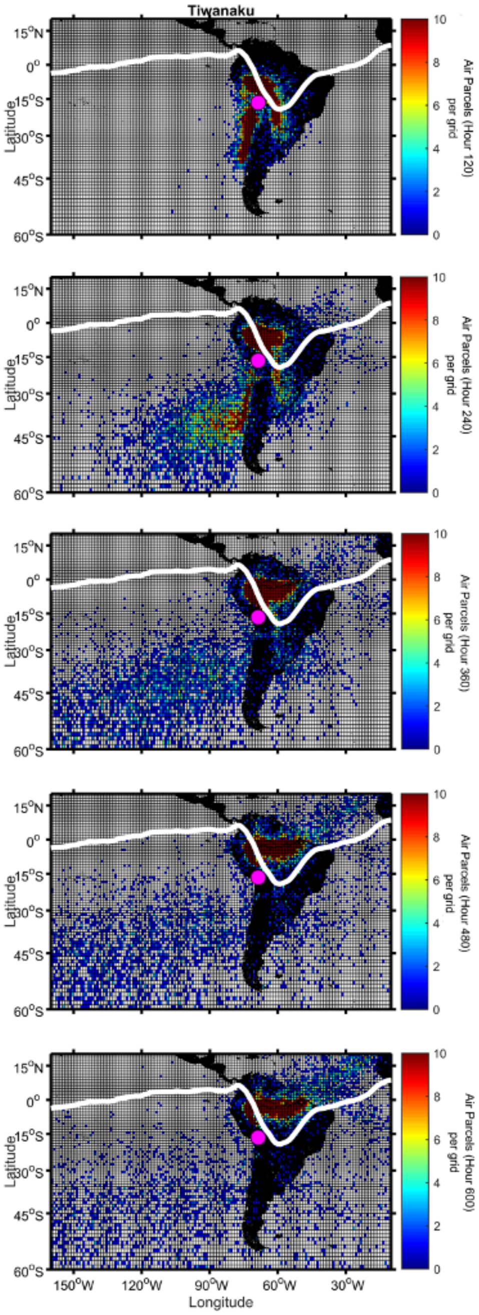

Fig S2.1. HYSPLIT backward trajectory analysis for Tiwanaku (the magenta dot). The colored scale indicates the number of air parcels located on each pixel grid (1×1°) at different times, one for each panel (hour -120, -240, -360, -480, and -600) during the study period (October–March, 1949–2019). The solid white lines show the average shape of the tropical low pressure belt that roughly marks the division between hemispheric air masses.

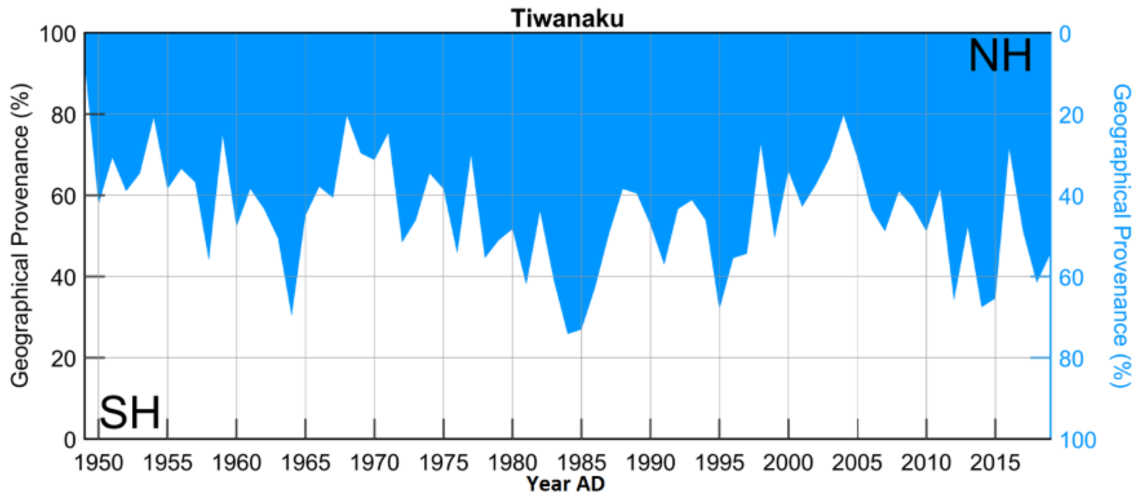

Fig S2.2. Air parcel contribution from each Hemisphere (%) at Tiwanaku (SH=Southern Hemisphere, white area; NH=Northern Hemisphere, blue area) for hour -600 (October–March, 1949–2019).

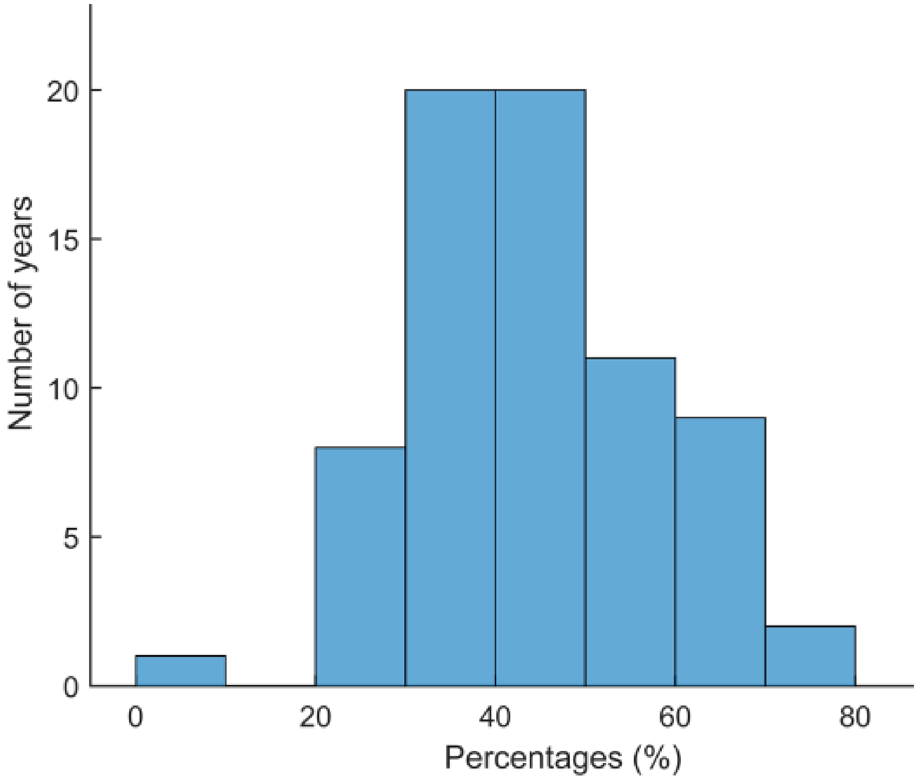

Fig S2.3. Histogram of Northern Hemisphere air parcels (%) at Tiwanaku during AD 1949–2019.

	October–March	November–March	November–February	October–February
Southern Hemisphere	55.64 ± 13.6	53.00 ± 14.9	53.77 ± 16.3	56.66 ± 14.5
Northern Hemisphere	44.35 ± 13.6	46.99 ± 14.9	46.22 ± 16.3	43.33 ± 14.5

Table S2.1. Comparison of relative hemispheric contribution (%) using averages for different sets of months, with and without November and February (hour -600). There are no significant differences between sets of months.

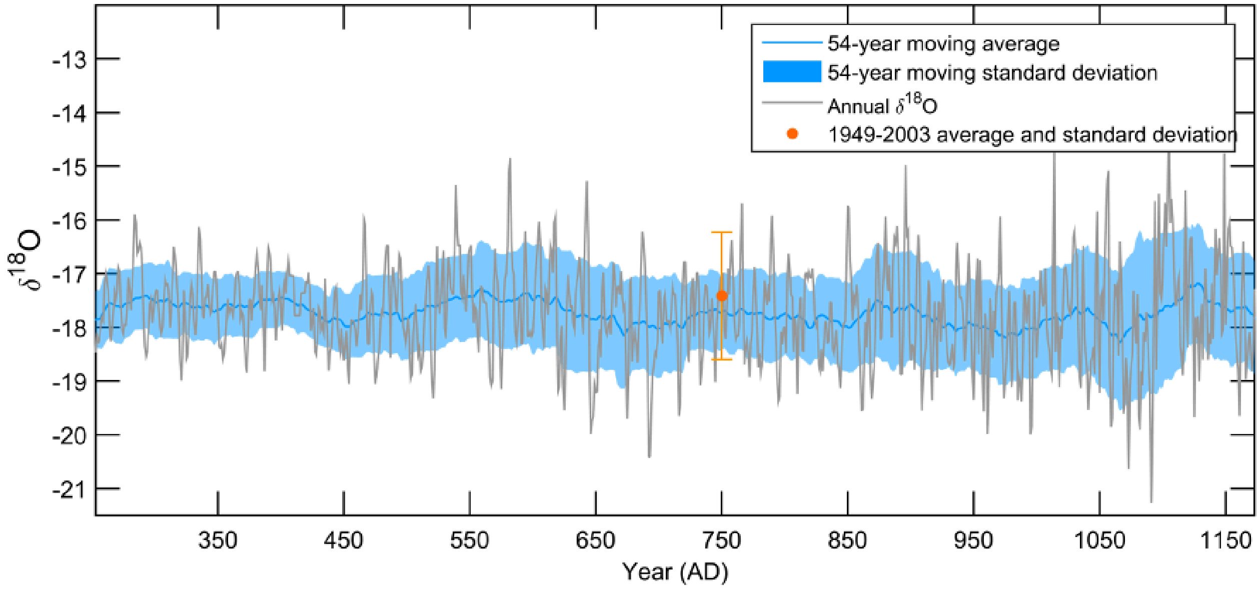

Fig S2.4. Annually-resolved $\delta^{18}O$ data from the Quelccaya ice cap, shown as the gray line. The blue line and shaded area represent the 54-year moving average and standard deviation. The orange dot and bracket represent the average and standard deviation for 1949–2003 (54 years, centered on AD 750).

In the case of Tiwanaku, the histogram of North Hemisphere input is a roughly normal curve (Fig S2.3), 55.6±13.7%, and passes all normality tests in PAST 4 [6]. Hence we could assume a mean and standard deviation, however, we suggest a more precise way of implementing this histogram that can be applied to areas with non-normal distributions. In OxCal, this can be done with an inline array that uses the values in the histogram: 1, 0, 8, 20, 20, 11, 9, 2, 0, 0. OxCal reads these as relative inputs, so it is not necessary to convert them to percentages. This inline array uses 100 values in brackets, set between a leading 0 and trailing 0. Coded this way, these 100 values reproduce the histogram that shows the amount of northern Hemisphere air parcels present at Tiwanaku (Fig S2.3).

```
Curve("IntCal20","IntCal20.14c");
Curve("SHCal20","SHCal20.14c");
Mix_Curves("Tiwanaku
mix","SHCal20","IntCal20",P(-1,101,[0,1,1,1,1,1,1,1,1,1,1,0,0,0,0,0,0,0,0,0,0,8,8,8,8,8,8,8,8,8,8,2
0,20,20,20,20,20,20,20,20,20,20,20,20,20,20,20,20,20,20,20,11,11,11,11,11,11,11,11,11,11,9,9,
9,9,9,9,9,9,9,9,2,2,2,2,2,2,2,2,2,2,0,0,0,0,0,0,0,0,0,0,0,0,0,0,0,0,0,0,0,0]));
```

These are reasonable estimates for the atmospheric carbon that organisms absorb over their lives, past and present. For short-lived samples, we cannot know the exact year the sample absorbed carbon, nor the atmospheric mixture of that year. This histogram represents the most likely situation. However, it is hypothetically possible that a short-lived sample could absorb 100% Northern Hemisphere air or 100% Southern Hemisphere air. If this uncertainty is an issue in future sampling efforts, we recommend the uniform mixed curve [1]. For most samples, including those in this paper, we think this inline array is most appropriate.

In practice, the inline array mixture produces results that are nearly identical to both a normal mixed curve of 56±14% (Table S2.1) and the unconstrained uniform mixture [1]. Differences were less than a decade, and often just 1–2 years, but this varies along the calibration curves. Some Tiwanaku dates fall near a calibration curve reversal. Around AD 750, atmospheric carbon levels returned to the same levels about eighty years prior (Fig S2.4). In the AD 800s, there was a short plateau. As our chronologies become more precise, wiggles in the calibration curve have a greater influence on the results. The most effective way to deal with this is with a modeled sequence of events and the use of bespoke site-specific mixed curves. Even though differences may be negligible in some places, Ancapichún et al.'s [2, 3] climate models are a small but firm step toward ever more precise archaeological chronologies. They will become increasingly relevant as our questions address individual life spans and, as we argue in this paper, rapid changes to past communities.

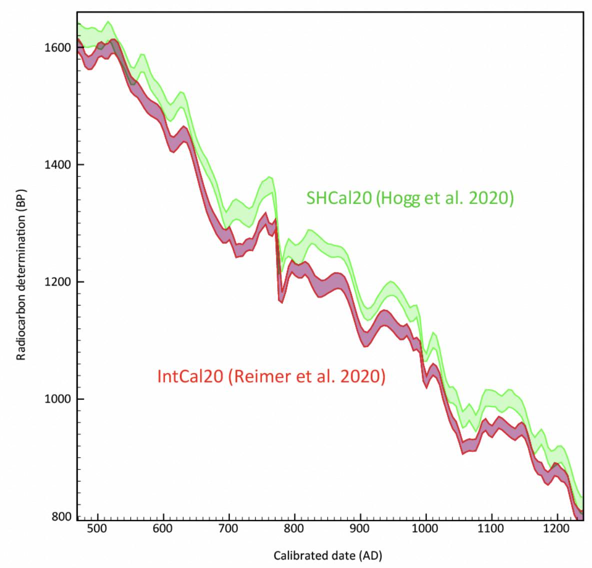

Fig S2.5. Wiggles in SHCal20 and IntCal20 for the centuries discussed in this paper. We mixed these calibration curves based on the modern mixture of air from both hemispheres at Tiwanaku.

Other considerations

In reporting dates, we follow established conventions [7, 8]. Italics indicate results from Bayesian models that depend on prior information; non-italic dates have only been calibrated. Medians of probability distributions are indicated with a tilde (~), and are followed by a date range and the corresponding probability. Since all dates are calibrated, we omit the usual abbreviation (cal). Dates from the same context are combined using the OxCal command Combine. Dates from the same individual are from the same carbon reservoir and hence combined with the command R_Combine, indicated with an ampersand (&). For this paper, we are ignoring possible minor temporal differences between dates on teeth from the same individual, since in many cases, the laboratory does not have a reliable record of which date applies to which tooth. We also disregard the lag time between the formation of a dated bone or tooth and the death date [9, 10]. These lags should be minor, since in most cases, the same individuals were dated with teeth that formed during childhood and none lived especially long. Future research should give this greater attention, as there is clear potential for precise chronologies of individual lives [11]. As an example of this, we provide a test case using dates from a female individual found in Ch'iji Jawira (CJ-35250 or TW099; S2 File) [12].

The dates from recent genetics papers are precise and include quality controls such as C:N and gelatin yield [13, 14]. Sampling human bone avoids the old wood problem, but tracking sample–artifact associations remains a key issue. Unfortunately, some recently-processed samples have

provided high precision duplicate dates and genomic information, but the lack of clear contextual details makes it difficult to incorporate these dates into Bayesian models and more generally, a robust narrative of the site's history.

When we speak of generation lengths, we are using 30 years as an estimate, rounding the estimates of 28 and 27 years from cross-cultural data and genomic patterns [15, 16].

The compilation of dates (S1 Table) is meant to be comprehensive for the site. We sought to correct errors from previous publications. Most of this is based on consulting original lab reports from the Wila Jawira project provided by John Janusek, building on previous efforts [16:230–231, 17]. For the dates from this project, Knobloch [17] provides a detailed review of the associated ceramics. In the main table for the project's dates, Janusek [18:Table 3.1] lists periods based on the median of the calibrated dates. These can be confused with the associated ceramics, which are taken from Janusek's unpublished notes [20]. We have excluded some of these from our list of contents with redwares, since they only have secondary associations without descriptions of the sherds [17]. In some cases, future research is required to clarify specific material associations, but these would not affect the overall results. For dates from other projects, details were often from unpublished excavation reports. This crucial information is often excluded from date lists, but it is essential to build reliable Bayesian models.

Janusek's [19] landmark ceramic seriation identified a series of substyles among Tiwanaku redwares. While some substyles tend to be earlier and others later, there is no clear correlation between substyles and phases. Janusek [18:82, 87] identified temporal shifts as assemblage-level tendencies and warned against attempts to create a master sequence, even though his analysis is sometimes used this way. Multiple substyles have been found in the same context, meaning they may reflect different types of contexts instead of temporal shifts, and these trends may not hold at all sites [21–25]. For this reason, we do not use ceramic substyles to build Bayesian models. Instead, we use the more general but much clearer ceramic distinction: Tiwanaku redwares, which includes all substyles. Visually, redwares are highly recognizable and clearly distinguishable from both other ceramics in the Andes and post-Tiwanaku styles. We note that in other regions, notably Moquegua, post-Tiwanaku ceramics are derived from Tiwanaku redwares. These are not present at Tiwanaku itself.

We do not include the site's initial set of dates processed in the 1950s and 1960s [19:Table 3.2, 26:Table 5, 27:206]. Most of these dates are from the previous Late Formative period, have poor information on artifact associations, or are from deep, mixed levels. Three of these are potentially relevant for the Tiwanaku period, based on artifact associations (P-119, P-121, and P-147) [27:55]. However, the samples were charred bone, and the dates were run before reliable pretreatment methods were developed. These are the only dates we manually rejected. Other dates are later than expected and have large error ranges, such as those in Akapana East, but these are not good reasons to manually reject dates [29]. Outlier models were not necessary and all models have acceptable agreement indices

(A_{model}>60% and $A_{overall}$>60%). The only significant assumptions are the groups and depositional sequences of dates. Overall, this suggests that the results are comprehensive, robust, and require few assumptions or quantitative manipulations. All other things being equal, adding new dates to these models should not significantly affect the boundaries, and most likely, adjustments will be within the error ranges (S2 File).

We used uniform boundaries for all models. In a few cases, trials with other boundary shapes showed higher agreement indices, but since this is not a good basis for choosing between competing, we use only uniform boundaries [29:192]. This is especially the case since most of the phases in this paper are populated by a small number of dates. There is no clear justification for using non-uniform boundaries, hence uniform boundaries should produce results that are "not importantly wrong" [7:7]. For sequential phases, we used First and Last queries instead of adding additional boundaries. While this has a negligible effect on the results, we avoided extraneous boundaries since they add unnecessary prior information into the model.

After running the main set of Bayesian models, we exported the calibrated posterior density functions (PDFs) for each context. Some contexts are dated with a single date; others are based on combinations of dates. The exported PDF files were modeled as single-phase models based on their material associations: Redwares, all contexts with human bone, tombs, violent deaths, and human bone not from tombs or violent deaths. The S1 Table lists these and other associations that did not inform the models. Many excavators describe offerings, but this is a category we found to be somewhat inconsistent so we did not use it to build Bayesian models. The OxCal code for all models is in the S3 File. The S4 File includes the full set of OxCal files, including processed files for each model, exported posterior PDFs, and a spreadsheet listing which model each was exported from.

References

1. Marsh EJ, Bruno MC, Fritz SC, Baker P, Capriles JM, Hastorf CA. IntCal, SHCal, or a Mixed Curve? Choosing a 14C Calibration Curve for Archaeological and Paleoenvironmental Records from Tropical South America. Radiocarbon. 2018;60: 925–940. doi:10.1017/RDC.2018.16
2. Ancapichún S, De Pol-Holz R, Christie DA, Santos GM, Collado-Fabbri S, Garreaud R, et al. Radiocarbon bomb-peak signal in tree-rings from the tropical Andes register low latitude atmospheric dynamics in the Southern Hemisphere. Science of The Total Environment. 2021;774: 145126. doi:10.1016/j.scitotenv.2021.145126
3. Ancapichún S, Pawlyta J, Rakowski AZ, Sieczkowska D. Influence of air parcels from Northern and Southern hemispheres on radiocarbon-based Inca chronology. Radiocarbon. 2022;64: 1431–1446. doi:10.1017/RDC.2022.87
4. Stein AF, Draxler RR, Rolph GD, Stunder BJB, Cohen MD, Ngan F. NOAA's HYSPLIT Atmospheric Transport and Dispersion Modeling System. Bulletin of the American

Meteorological Society. 2015;96: 2059–2077. doi:10.1175/BAMS-D-14-00110.1
5. Thompson LG, Mosley-Thompson E, Davis ME, Zagorodnov VS, Howat IM, Mikhalenko VN, et al. Annually Resolved Ice Core Records of Tropical Climate Variability over the Past ~1800 Years. Science. 2013;340: 945–950. doi:10.1126/science.1234210
6. Hammer Ø, Harper DAT, Ryan PD. PAST: Palaeontological Statistics Software Package for Education and Data Analysis. Palaeontologia Electronica. 2001;4: 1–9.
7. Bayliss A, Bronk Ramsey C, van der Plicht J, Whittle A. Bradshaw and Bayes: Towards a Timetable for the Neolithic. Cambridge Archaeological Journal. 2007;17: 1–28. doi:10.1017/S0959774307000145
8. Millard A. Conventions for Reporting Radiocarbon Determinations. Radiocarbon. 2014;56: 555–559. doi:10.2458/56.17455
9. Ubelaker DH, Thomas C, Olson JE. The impact of age at death on the lag time of radiocarbon values in human bone. Forensic Science International. 2015;251: 56–60. doi:10.1016/j.forsciint.2015.03.024
10. Ubelaker DH, Plens CR, Soriano EP, Diniz MV, de Almeida Junior E, Junior ED, et al. Lag time of modern bomb-pulse radiocarbon in human bone tissues: New data from Brazil. Forensic Science International. 2022;331: 111143. doi:10.1016/j.forsciint.2021.111143
11. Millard AR, Annis RG, Caffell AC, Dodd LL, Fischer R, Gerrard CM, et al. Scottish soldiers from the Battle of Dunbar 1650: A prosopographical approach to a skeletal assemblage. PLOS ONE. 2020;15: e0243369. doi:10.1371/journal.pone.0243369
12. Becker SK. Skeletal evidence of craft production from the Ch'iji Jawira site in Tiwanaku, Bolivia. Journal of Archaeological Science: Reports. 2016;9: 405–415. doi:10.1016/j.jasrep.2016.08.017
13. Nakatsuka N, Lazaridis I, Barbieri C, Skoglund P, Rohland N, Mallick S, et al. A Paleogenomic Reconstruction of the Deep Population History of the Andes. Cell. 2020;181: 1–15. doi:10.1016/j.cell.2020.04.015
14. Popović D, Molak M, Ziółkowski M, Vranich A, Sobczyk M, Vidaurre DU, et al. Ancient genomes reveal long-range influence of the pre-Columbian culture and site of Tiwanaku. Sci Adv. 2021;7. doi:10.1126/sciadv.abg7261
15. Fenner JN. Cross-cultural estimation of the human generation interval for use in genetics-based population divergence studies. American Journal of Physical Anthropology. 2005;128: 415–423. doi:10.1002/ajpa.20188
16. Wang RJ, Al-Saffar SI, Rogers J, Hahn MW. Human generation times across the past 250,000 years. Science Advances. 2023;9: eabm7047. doi:10.1126/sciadv.abm7047
17. Knobloch PJ. Tiwanaku's Coming of Age: Refining Time and Style in the Altiplano. In: Vranich A, Stanish C, editors. Visions of Tiwanaku. Los Angeles: Cotsen Institute of Archaeology, University of California; 2013. pp. 211–233.
18. Owen B. Distant Colonies and Explosive Collapse: The Two Stages of the Tiwanaku Diaspora in the Osmore Drainage. Latin American Antiquity. 2005;16: 45–81. doi:10.2307/30042486
19. Janusek JW. Vessels, Time, and Society: Toward a Chronology of Ceramic Style in the Tiwanaku Heartland. In: Kolata AL, editor. Tiwanaku and Its Hinterland: Archaeological and Paleoecological Investigations of an Andean Civilization, Vol 2: Urban and Rural Archaeology.

Washington, D.C.: Smithsonian Institution Press; 2003. pp. 30–92.
20. Marsh EJ, Roddick AP, Bruno MC, Smith SC, Janusek JW, Hastorf CA. Temporal Inflection Points in Decorated Pottery: A Bayesian Refinement of the Late Formative Chronology in the Southern Lake Titicaca Basin, Bolivia. Latin American Antiquity. 2019;30: 798–817. doi:10.1017/laq.2019.73
21. Alconini Mujica S. Rito, símbolo e historia en la pirámide de Akapana, Tiwanaku: un análisis de cerámica ceremonial prehispánica. La Paz, Bolivia: Acción; 1995.
22. Augustine JMF. Style, Aesthetics, and Politics: Polychrome Ceramic Iconography in the Tiwanaku Valley, AD 500-1100. Ph.D., The University of Chicago. 2019. Available: http://search.proquest.com/docview/2311072388/abstract/839A4A82457B423FPQ/1
23. Burkholder J. Tiwanaku and the Anatomy of Time: A New Ceramic Chronology from the Iwawe Site, Department of La Paz, Bolivia. Department of Anthropology, State University of New York. 1997.
24. Korpisaari A. Death in the Bolivian High Plateau: Burials and Tiwanaku Society. Oxford: British Archaeological Reports; 2006.
25. Rivera AF. Espacios ceremoniales al pie de Akapana: excavaciones de las unidades N2043-E1023 / N2043-E1024. Licenciatura Thesis, Pontificia Universidad Católica del Perú. 2011.
26. Ponce Sanginés C. Las culturas de Wankarani y Chiripa y su relación con Tiwanaku. La Paz: Academia Nacional de Ciencias de Bolivia; 1970.
27. Marsh EJ. A Bayesian Re-Assessment of the Earliest Radiocarbon Dates from Tiwanaku, Bolivia. Radiocarbon. 2012;54: 203–218. doi:10.2458/azu_js_rc.v54i2.15826
28. Ralph EK. University of Pennsylvania Radiocarbon Dates III. American Journal of Science Radiocarbon Supplement. 1959;1: 45–58. doi:10.1017/S003382220002035X
29. Hamilton WD, Krus AM. The Myths and Realities of Bayesian Chronological Modeling Revealed. American Antiquity. 2018;83: 187–203. doi:10.1017/aaq.2017.57
