# Supplementary material for: The center cannot hold: A Bayesian chronology for the collapse of Tiwanaku
Source: PLoS One. 2023 Nov 22;18(11):e0288798. doi: 10.1371/journal.pone.0288798 (PMC10664893; doi:10.1371/journal.pone.0288798)
Supplement: S2 File — (PDF) [file pone.0288798.s003.pdf]

Supplementary file for:

Marsh EJ, Vranich A, Blom D, Bruno M, Davis K, Augustine J, et al. The center cannot hold: A Bayesian chronology for the collapse of Tiwanaku. PLOS ONE. 2023;18: e0288798. doi:10.1371/journal.pone.0288798

S2. Description of depositional sequences and Bayesian models

This supplementary document provides details on the archaeological contexts of the radiocarbon dates, which are the basis for the assumptions in the Bayesian models.

Tiwanaku was founded in the second century AD during the Late Formative Period [1, 2]. During this period, we have evidence of a few residential contexts and two major constructions, The Sunken Temple and the Kalasasaya. The Kalasasaya has early burials and dates and near the modern surface, including a recently-excavated cache of Qeya vessels, which are diagnostic of the Terminal Late Formative [2, 3]. All of these contexts are earlier than those discussed in this article. Here, we discuss the principal occupation of Tiwanaku, when there was a resident population using decorated redwares, roughly AD 600–1000 [4]. Within this period, excavators have identified early and late phases, sometimes tied to the ceramic styles Tiwanaku IV and V, but we do not use these styles in the Bayesian models (see S1 File). Later, centuries after the city's collapse, there is a small Inca period occupation best documented around the Pumapunku and north of the modern town [5–7; see Table 1].

Here we summarize excavation details, with more detailed treatments of unpublished data. Bayesian models were built based on depositional sequences in each area and then cross-referenced into composite models.

Akapana East, Muru Ut Pata, and Ch'iji Jawira

East of the Akapana, excavations have produced 19 dates associated with residential occupations in five nearby excavation areas: Akapana East [8–12], Muru Ut Pata, and Ch'iji Jawira (Fig S2.1; see [13] for updated maps with geophysical survey results and other excavations areas). For many of the contexts mentioned below excavated as part of project Wila Jawira, field forms and photographs have been made available online [14].

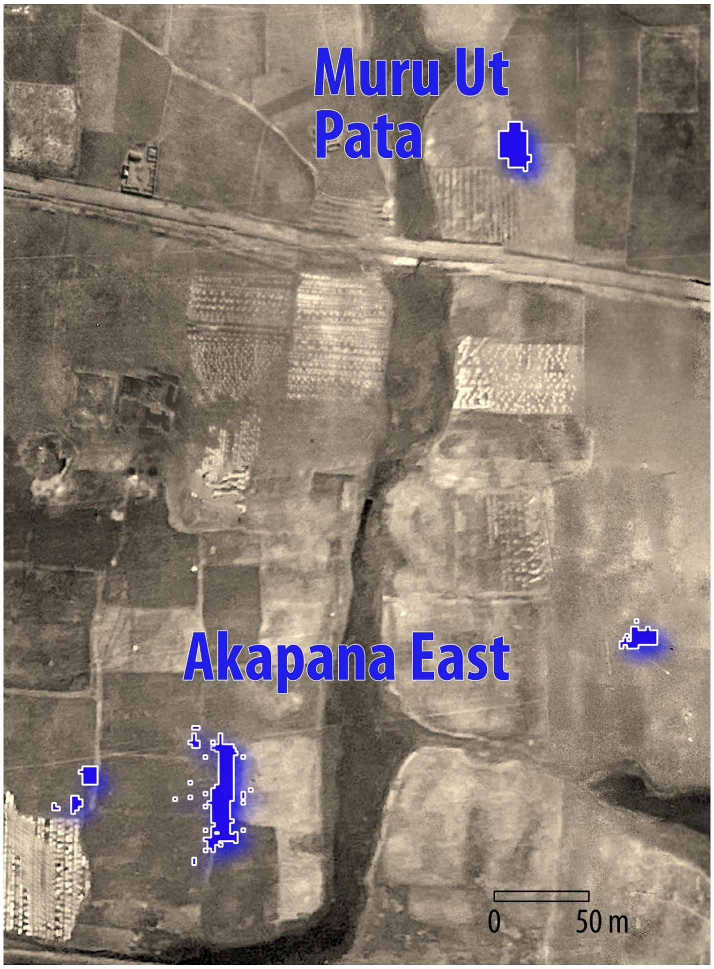

Fig S2.1. Detail of Muru Ut Pata (Akapana archaeological project) and Akapana East (project Wila Jawira), showing excavated units in blue. See overview map in Fig 1. The basemap is a 1930s aerial orthomosaic (available from https://osf.io/v6j7n/; see S5 File). The locations of excavation units from project Wila Jawira are from [14]. This project labeled units based on their southwest corner on an arbitrary grid. The grid overlays onto UTM zone 19 with the origin at 529829 m E, 8161529 m S. The project's feature layers can be loaded into GIS software as ArcGIS REST service layers using links from this page (this list also includes layers from other OCHRE projects): https://services1.arcgis.com/d51RD4kgsa1HoRoy/ArcGIS/rest/services

Akapana East

There are 15 dates from three excavated areas in Akapana East, which we combined into a single Bayesian model.

First, there are five dates from Akapana East 1 Mound. Tiwanaku period occupations include residential structures with dense kitchen refuse and early Tiwanaku IV ceramics [9:128–138, 441, Fig 7.12, 10:269–270]. An early surface was dated to ~AD 570 (Beta-55491, Structure 2). There is an infant burial from within an early wall foundation, but the error range of ±276 makes it

uninformative (SMU-2333) [9:441]. The area includes extensive domestic refuse, ad hoc drainage ditches, large storage pits and, in one outdoor patio, ten superimposed surfaces. This suggests permanent occupation in the first part of the Tiwanaku period. The area was abandoned and then reoccupied sometime later [9:138].

This area has also a clear case of late, temporary occupation. Small refuse pits cut into earlier structure foundations. Unusually, this late temporary occupation did include a small structure, with reused stone set in a different configuration than was common in the prior Tiwanaku Period (see photographs [14]; for map and ceramics, see [15:Fig 10.6a]). It was perhaps for seasonal occupation, and had mostly domestic refuse in addition to Tiwanaku keros and tazones [15:189] (in this supplementary file, we use Tiwanaku-specific terms for decorated ceramic vessels: keros are serving goblets; tazones and escudillas are serving bowls [4, 16]). Two nearby dates are late and have unwieldy error ranges of ±115 and ±197 years (SMU-2332 and SMU-2331) [9:177–178, 15:191]. Farther east, closer to the site's perimeter canal, there was a similar pattern of refuse accumulating over melted wall foundations, implying that some time had passed between the abandonment of the structure and its later more ephemeral occupation. There was a midden with "only Tiwanaku-style artifacts" *~AD 1230* but this date has an error range of ±183 years (SMU-2470) [15:190]. In another nearby structure, there are Early Pacajes ceramics found with rare examples of transitional Tiwanaku–Pacajes style ceramics, though these contexts are undated [9:377].

Second, there are eight dates from Akapana East 1. Horizontal excavations in Akapana East 1 exposed clean surfaces, secondary burials, and a rare Qeya sherd, a ceramic style that is diagnostic of the Terminal Late Formative [3:106–108, 9]. Above this, surfaces include Tiwanaku IV ceramics and a date with a median that falls in the early Tiwanaku period *~AD 660* (SMU-2471) [9:108]. A slightly later date comes from a cist-burial in a defined mortuary area *~AD 730* (AA-107585) [9:308, 445]. The final occupation zone is 3–10 cm deep with dense domestic refuse over an irregular surface and no prepared floors. Diagnostic ceramics are in the Tiwanaku V style. One date is from the surface near structure 1 (SMU-2469) and the other five are from ashy pits filled with domestic refuse (SMU-2277, SMU-2278, SMU-2289, SMU-2290, SMU-2276) [9:302–306]. These six dates have very similar medians of *~AD 840–940*. Like elsewhere in the sector, the larger pits were probably initially made to extract clay for walls, construction fill, and ceramics.

Third, there is one date from Akapana East 2. Here, Tiwanaku occupations overlie sterile soil. A single date comes from an unusual three-chambered burial, *~AD 840* (AA-107586) [9:144–145, 444, Fig C3, 11:155]. The sample comes from the north chamber, which included bones of a 3–4-year-old child, a decorated tazón and an escudilla. Covering the surface associated with the burial is domestic refuse followed by a charred roof thatch, marking its abandonment [8:140–148, 9:251]. Finally, people dug pits that cut into previous occupations.

In the Bayesian model for Akapana East, we built all 15 dates in a Bayesian model with three phases. The early phase includes three dates from the Tiwanaku IV occupations of Akapana East 1 Mound and Akapana East 1, with modeled medians of *~AD 630–650*. Next, two burials have medians of *~AD 730* and *850* (AA-107586, AA-107585), but the model does not assume during which phase they were placed since stratigraphic relationships are not clear. There are no occupational dates with medians in the AD 700s, hinting that the sector might have been abandoned for a few generations. The middle occupation phase is the best defined, with abundant material and six radiocarbon dates, grouped as a phase [9:138]. This phase probably spanned *~AD 860–950*, the first and last modeled medians. The most important result is the depositional boundary after the second phase of occupation, since it marks the collapse of building walls and the end of permanent residence *~AD 1020 (910–1140, 95%)*. The final occupation is characterized by temporary occupations, and is defined as a phase of three dates from Akapana East 1 Mound. Their medians span *~AD 1100–1160* and error ranges are very wide (±115–183). Refuse accumulated over

collapsed adobe structures suggests at least a generation passed before visitors reoccupied the area [15:441, 16:189]. The associations between the dates and the ceramics are not especially clear, complicated by intrusive pits and post-depositional processes. Since these are temporary reoccupations, it is quite feasible that visitors reused old structures and even mixed their own refuse with earlier deposits, including earlier decorated ceramics.

Muru Ut Pata

Muru Ut Pata is a low mound measuring about 50×50 m, located north of Akapana East 2, just outside the perimeter canal (Fig S2.2). These are unpublished excavations from two field seasons in 2005 and 2006, which exposed a contiguous area of 326.5 m^2 labeled sector R. Several episodes of construction and modification of structures and spaces were identified, but carbon dating and ceramic analysis indicates that this area was occupied only at the end of the Tiwanaku Period. Two deep excavations revealed no evidence of occupation prior to the dated contexts.

In the northwest section of the excavation area, several small structures open onto a patio space partially surrounded by a thicker, compound wall. These structures, defined by the remains of double rows of cobblestone adobe wall foundations, are cell-like rooms with interiors of around 2.5×2.5 m. Canals and conduits run south-to-north, down the natural decline of the mound, located in the westernmost part of the sector in the patio area. Inside three structures, there were circular hearths with evidence of burning in depressions on the occupation surface. Ceramics include cooking and storage vessels as well as an abundance of decorated Tiwanaku redwares such as keros, puma-head incense burners, and tazones. There are rare non-local ceramics, including greyware from Sucre or Chuquisaca and from Cochabamba. Faunal analysis indicates that residents of Muru Ut Pata enjoyed first-hand access to whole camelids. They butchered them here and made tools from the bones.

As in other residential spaces, occupation surfaces were pock-marked with ash pits and middens. One was dated, which is stratigraphically associated with the main occupation of the sector (Unit 13, level 8, UGAMS-26434). A number of shaft tombs were probably placed around the time of the sector's residential occupation and the individuals in them tended to be older. This follows the site-wide pattern of placing the dead in residential areas. One shaft tomb included a kero and charcoal from inside the vessel was dated (feature 11, Unit 10, level 14, UGAMS-26433). These two dates were modeled as a phase, since the depositional sequence between them is not clear. The model places both dates at *~AD 920 (890–990, 95%)*. The last dated event is from a layer of organic remains immediately overlying the occupation surface, which places the abandonment of the sector close to *~AD 950 (900–1000, 95%;* Unit 10, level 7, UGAMS-26432). Since this model is based on only three dates, the phase boundaries are not especially informative. The very similar medians suggest a brief residential occupation.

After the sector was abandoned, burials were placed close to the surface without the burial architecture of a shaft tomb. There is no clear evidence of a violent death, but these remains may be an example of a violent death, which are mostly young individuals (Fig S2.3). The other sets of superficially placed adult remains are laid in fetal positions on their sides or on their backs tightly flexed with a vessel. These individuals were often placed with a single vessel. A kero and a blackware vessel, were included in two cases, both of which were chipped and carefully modified, suggesting they were reclaimed from older contexts and repurposed. These late contexts have not been dated.

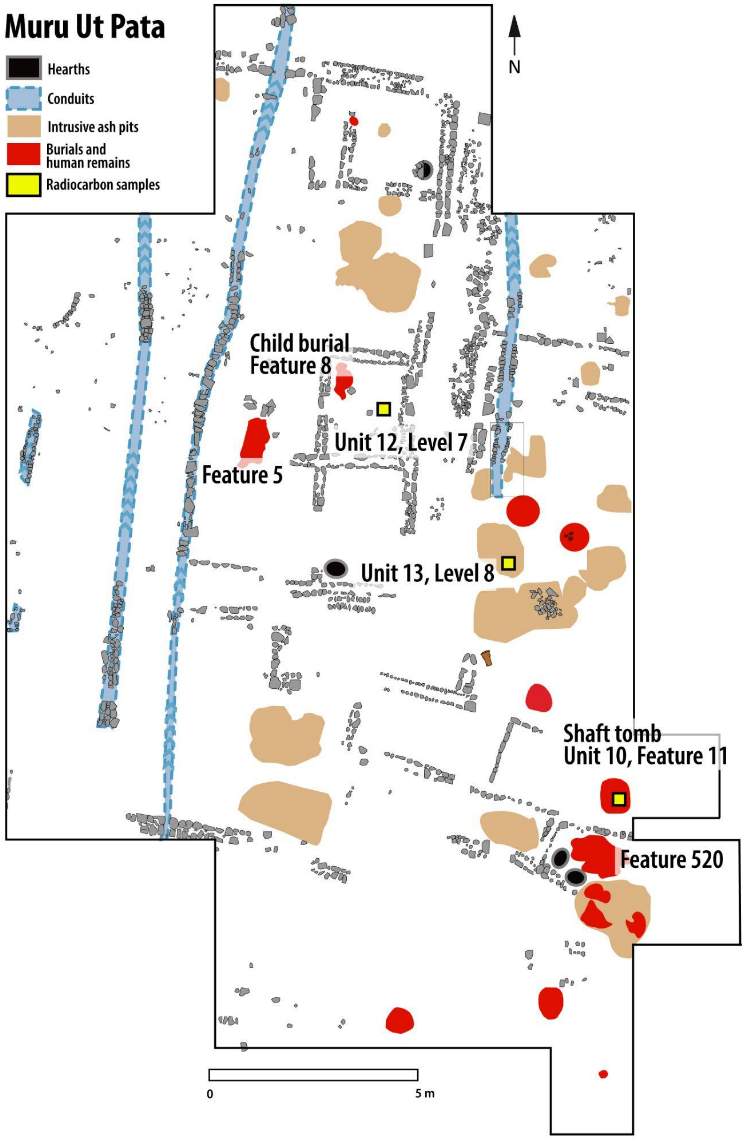

Fig S2.2. Plan drawing of major features from the excavation of Muru Ut Pata, indicating the location of radiocarbon samples and features mentioned in the text.

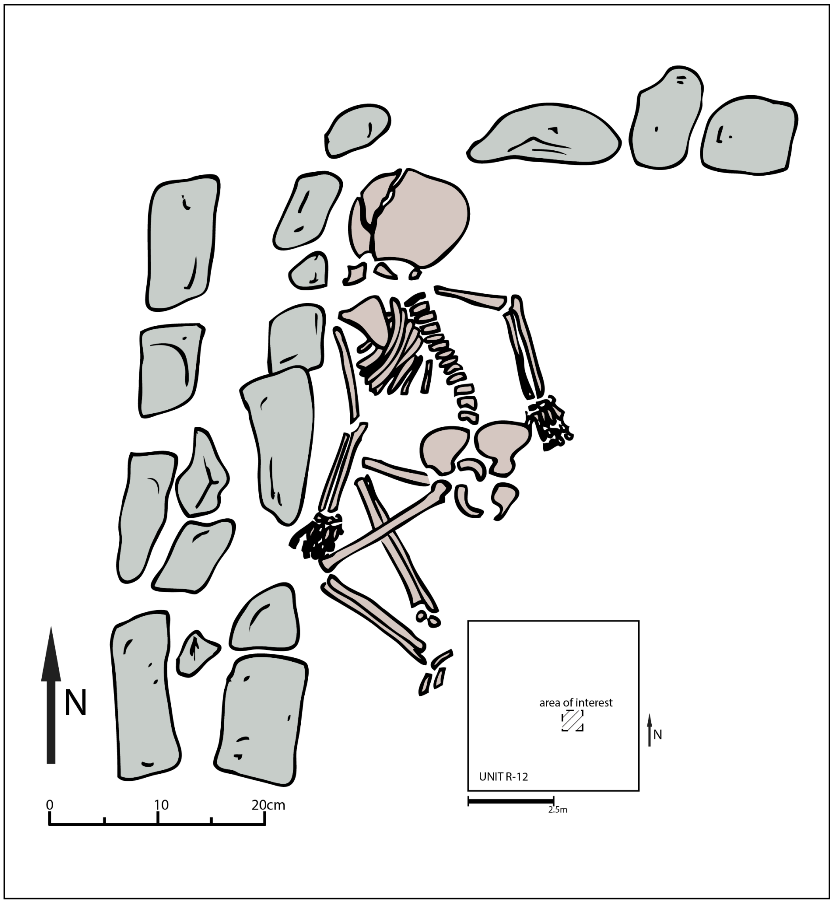

Fig S2.3. Child burial from Muru Ut Pata (Feature 8), located away from the cluster of burials to the southwest (Unit 12, Zone R, Level 9). This skeleton is of a 4–6-year-old, has clear fronto-occipital cranial modification, and the craniofacial bones are fractured, though it is not clear if this is perimortem trauma or post depositional. Near the feet, there was a finely-worked basalt tube, possibly an earspool. This burial's location and informal treatment differ notably from other burials, for example, Features 5 and 520 (see Fig S2.5). The general impression is that the child was left unceremoniously on the floor in the corner of a room.

Ch'iji Jawira: two dates from one well-studied woman

Ch'iji Jawira has a series of Tiwanaku period burials and residential areas that were probably inhabited by potters [8, 17:146–147, 160] (this area is located east of the maps shown in this article). Potting may have been a principal activity during the life of a 30–39-year-old woman buried in this sector, based on skeletal indicators (individual CJ-35250 or TW099) [19]. Two teeth were sampled, the sector's only radiocarbon dates, which combine and calibrate to ~AD 720 (660–830, 95%; TW099; Poz-122665 & Poz-122666). Wiggle-matching the dates and her estimated age at death suggests she was alive ~*AD 710–750*. However, this model is preliminary since the laboratory could not confirm which date was on which tooth. This approach follows [20] based on the ages of eruption for each tooth [20:Table 11]. Dietary isotopes are similar to other Tiwanaku-period individuals [21:237]. She was found with articulated camelid bones, an undecorated jar, and a gold lamina with an embossed face [14:298] (see photos in [17], compare a similar lamina from the Putuni

in [22:Fig 9.41]). These items initially suggested a Late Formative date for this burial, but these radiocarbon dates place it firmly in the Tiwanaku period, bringing to mind scenarios of heirloom items or nearly-forgotten vessel forms.

This burial was one of the earliest depositional events in Ch'iji Jawira, so it was probably placed around the time of the first residential occupation [11:160–161, 17:298]. This means most of the sector's cultural material was deposited after this potter's death *~AD 750*. This is centuries later than once thought, meaning these ceramists were not involved in the initial population surge at the site. During the early part of the Tiwanaku Period occupation, ceramic production may have been organized informally or embedded within families, perhaps similar to Late Formative patterns [24].

Mollo Kontu

Mollo Kontu Mound

The Mollo Kontu mound (also called sector M) is located south of the Akapana and measures 50×40×3.5 m (Fig S2.4). Excavations of 25 m^2 in the 1950s identified 30 burials [25]. Later, 1990–1991 excavations of 224 m^2 clarified that the mound originally had terraces and walls, recalling the Akapana, which was also where many burials and offerings were placed. However, unlike the Akapana, there was no standing architecture or formal access points [25:212, Fig 8.22]. Many burials were placed along the mound's main revetment. The low frequency of drinking vessels suggests this was not a location for ritual feasting but instead, activities focused on dedicatory internments for individuals of all ages [25:218]. Ceramic patterns suggested that this area dated to the earlier part of the Tiwanaku period [25:216], but the radiocarbon dates indicate that the burials were placed later. There are eight dates on seven individuals, all from the latest part of the site's occupation. One date is from a 15–20-year-old who was buried with an infant ~AD 1000 (890–1030, 95%, AA-107601); there were no grave goods but there were textile impressions (Feature 10, MK-34967) [25:212, 218]. The second context included at least seven individuals. The dated individual is a 35–45-year old probable male who was found face down with his hands bound behind his back (Feature 19, MK-39787) [25:221, Fig 8.37]. The date is ~AD 990 (890–1030, 95%; AA-107602).

In 2007, excavations of 96 m^2 identified nine individuals, who were all found along the base of the north wall that defines the mound [27]. Four of the individuals were dated but there were no clear stratigraphic relationships between them [26:33–34, Figs 3.8–13, 3.19]. Two died around the same time as many other burials at Tiwanaku: a 4–5-year-old child ~AD 1090 (990–1160, 95%; MK-04599, AA-107597) and a 45–49-year-old woman ~AD 940 (880–1000, 95; MK-04583, AA-107596). The other two died centuries later: an infant ~AD 1250 (1210–1280, 95%; MK-04600, AA-107598), and a young female ~AD 1300 (1270–1390, 95%; MK-04611, AA-107599). This adult was buried with a bone weaving tool; strontium isotopes suggest she was non-local. These seven dated individuals suggest relatively late use of the area: four have medians of ~AD 940–1000 and another three, ~AD 1090–1300. The latest dates suggest that the Mollo Kontu mound remained a long-term location for leaving human offerings and interments.

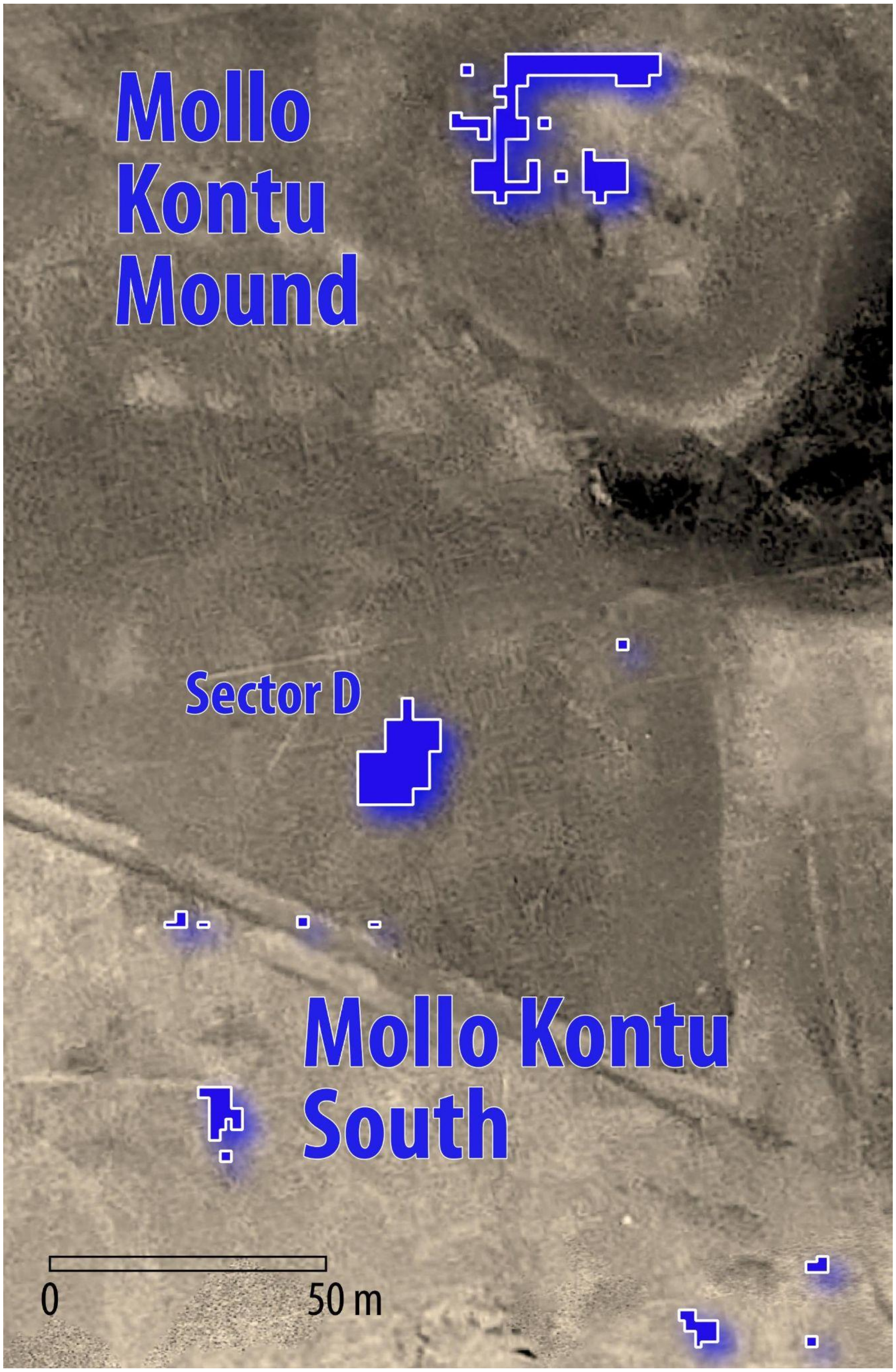

Fig S2.4. Excavated units in Mollo Kontu by projects Wila Jawira and Jach'a Marka. The mound (sector M is in the north) and the southern area, with sector D is in the center of the image and most other smaller sectors farther south.

Mollo Konto South

Mollo Kontu South is located south of the mound (Fig S2.4) [28]. The first excavations here were conducted in 1990 and 1991 [26]. These were followed up by a large project, Proyecto Jach'a Marka, that excavated two areas: 1) sectors A, B, C, and F and 2) sectors D and G [29–32].

The principal finds in sectors A and C were human burials. In sector C, 2001 excavations of 47 m^2 identified a number of burials. Four dates were run on three individuals, all from unit 5 (Features 12, 19, and 22) [32]. In this sector, two burials were found with gold laminas (Feature 12, MK-00731 and in feature 21, MK-00874-1); other grave goods included obsidian projectile points and a beaded necklace. All three dated individuals died around the same time, with medians of ~AD 570–590, which are among the earliest dates from Tiwanaku's main occupation.

In sector A, excavators identified a series of burials that cut into four distinct surfaces, each of which corresponded to a period of occupations labeled I–IV. Originally considered a specialized cemetery, it seems more likely sector A was a disturbed residential area with more burials than other residential areas, such as sector D. Since the residential materials and their relationships with the dated burials are not completely clear, we excluded sector A burial dates from the residential models. These early dates are less important for defining the collapse chronology, but future research on Early Tiwanaku should give sector A's residential occupations fuller consideration. Six sector A contexts, five of which were burials, were dated. The earliest dated burial (event A91) corresponded to the second oldest occupation (III), which was located around 40 cm below the contemporary surface. The ceramic sherds from this context appear to pertain to refuse as opposed to funerary offerings. A charcoal sample from event A91 produced a date of *~AD 680 (640–770, 95%*; AA-275873; a long bone sample from individual MK-05409 failed for lack of collagen). The second dated grave (A34) contained an individual who was interred without grave goods during occupation II *~AD 710 (650–780, 95%*; AA-175874; A34). Cutting into the occupation I surface, the final two burials were rectangular chambers containing young individuals with poorly preserved bones. These tombs were capped with a stone (A96–97) and an adobe (A102–103) block, respectively, which possibly facilitated repeated access to their inhabitants and contents (Fig 5a–c). At the base of burial A96, a small offering (A99) had been placed, containing a tazón, an incense burner, and a portrait vessel (Fig 4b, c; Augustine 2019:Fig 6.25). Dates from these infants (individual (MK-05144 and MK-05404) suggest they died around the same time: *~AD 760* and *~AD 790 (690–890, 95%*; AA-275871, AA-275872).

At approximately 20 cm below the surface, a large midden (A111), located more-or-less within the plow zone, was dated to *~AD 1040 (990–1150, 95%, Beta-275875)*. Excavators recovered animal bones and ceramics, including kero sherds (Fig 4a). Finally, the bones of a 9–11-year-old child were found buried with a kero and a tazón; however, this burial has no clear stratigraphic relationships with the other occupations (MK-3215) [32:2–4]. Radiocarbon dates indicate this individual died much later than the other individuals found in sector A: two tooth dates combine and calibrate to ~AD 970 (890–1030, 95%; TW110: Poz-122713 & Poz-122714).

In sum, sector A was a heavily distributed residential area where a number of burials were placed. Of the five dated individuals, four have medians spanning *~AD 680–790*, followed by a later burial ~AD 970 that is contemporaneous with burials from the Mollo Kontu mound. The burials in sector A mostly correspond to the first two centuries of Tiwanaku's main occupation. The current evidence suggests that the mound superseded sector A as the focal point of funerary activity after around the ninth century AD.

Residential occupation in sector D

Between 2005 and 2008, excavations in sector D uncovered a large expanse of residential space, approximately 180 m^2, in Mollo Kontu South (Figs S2.5 and S2.6) [33:70–92, Table 3.1, 34:163–171]. These excavations produced the best currently available data for residential occupation within the area. Excavators identified four more-or-less distinct occupations (from earliest to latest, V, IV, III, and I–II), based on stratigraphic relationships between compound walls, refuse pits, and hearths. However, certain features were grouped into a transitory phase, called IV/III, which corresponds to a period of urban renewal at Mollo Kontu, during which earlier residential spaces were replaced with new compound walls and living quarters. These occupations are arranged into a sequence of three phases in the Bayesian model: first V, then IV–III, and finally I-II.

The excavations in sector D sought to uncover a large horizontal area of residential features. As a result, nowhere did excavators reach sterile soil, and we do not have a good sense of when the sector was first occupied permanently. Only a small snapshot (approximately 16 m^2) of the earliest occupation (V) was uncovered. At this depth, the remains of a large north–south compound wall were identified, along with three relatively small pits. Two samples from this occupation were radiocarbon dated. The first is burnt wood recovered via flotation from the compound wall's foundation, which dates its original construction, *~AD 680 (600–770, 95%; Beta-275868)*. The second sample comes from one of the aforementioned small middens, *~AD 750 (630–780, 95%; Beta-275869)*. In the Bayesian model, this date's agreement index is low, but we retain it in the model because the stratigraphic relationship is clear, as the pit forming the midden cut into adobe melt from the earlier compound wall [30:76]. While occupation V is roughly contemporaneous with the earliest occupations of other sectors of Tiwanaku, we cannot rule out the possibility that Mollo Kontu South was settled prior to this stratum.

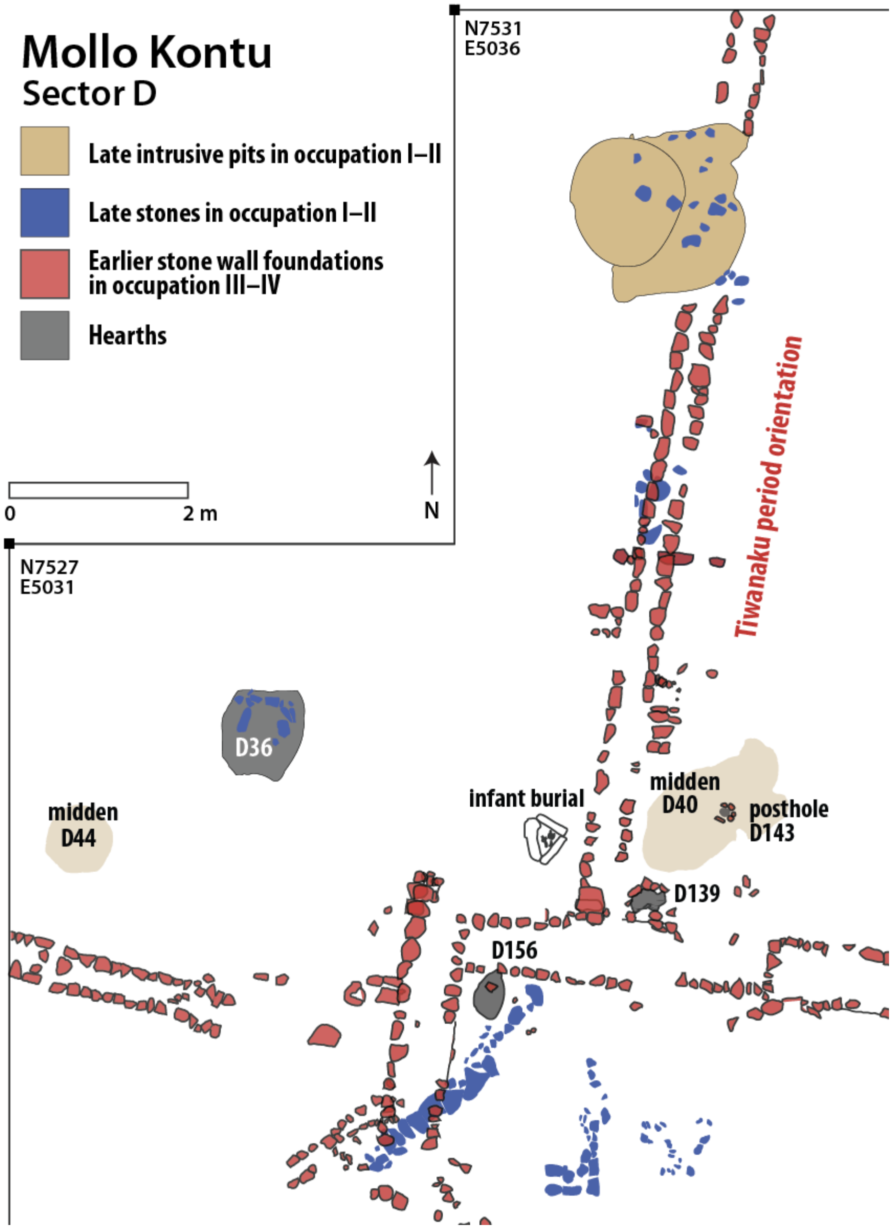

Fig S2.5. Excavation detail of Mollo Kontu, sector D, with labels for dated depositional events. Stone wall foundations from occupation III and IV are in red, which follow the site-side orientation of 6–8° east of north during the Tiwanaku period [8:150]. Later stones from occupation I–II are in blue, which have a different orientation. Intrusive pits suggest later visitors reused adobe and stone from earlier buildings. Solid black lines show the northern and western edge of excavations, with two corner coordinates from the site-wide grid.

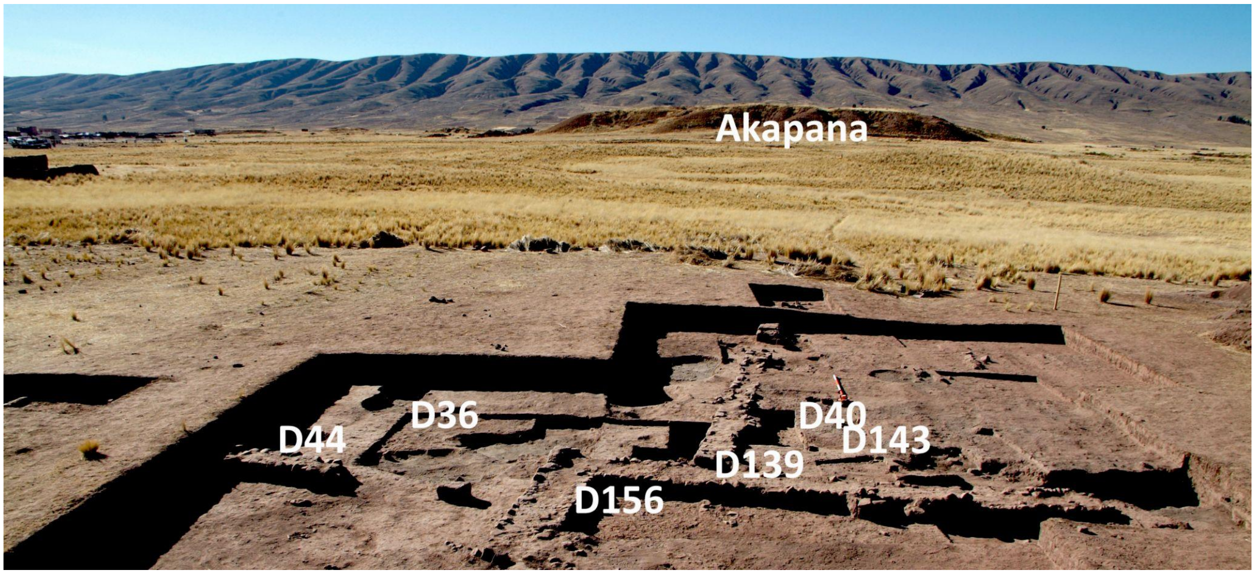

Fig S2.6. Mollo Kontu sector D excavations, looking north. Dated depositional events are labeled. Photograph by Jeff Clarke, used with permission.

The second and third occupations (III–IV) were more extensively excavated than occupation V. From these strata, better-preserved walls were identified and five radiocarbon dated samples were obtained. The key set of features that characterized occupation IV was a network of interconnecting compound and room walls. These walls demarcated the excavated area into three spaces. The western space was made up of middens and an open activity area. Excavators considered both the northeast and southeast spaces to be indoor cooking and living quarters. Occupation III was characterized by a series of large amorphous pits, some of which cut into the northeast living quarters of occupation IV, as well as a new east–west compound wall in the previously open western section of the excavations. These pits and hearths respect the walls, suggesting residents made them while the walls were still up and conditioned the use of space. The same is true of an infant burial (events D114, 122–125) [33:77, 84]. While excavators inferred stratigraphic relationships between features in the three spaces of sector D, there remains uncertainty about the precise depositional sequence of all features.

Of the five dated contexts, three were located in the northeastern space. Feature D143 was a cylindrical hole near the intersection of the main north–south and east–west compound walls of occupation IV. Excavators provisionally identified this feature as a posthole; however, since no other postholes were identified in Mollo Kontu South, it is difficult to say what it was for certain. The second feature within the northeastern space from which a dated sample was taken is D139. D139 was a hearth that was stratigraphically superior to D143 [30:77], and this relationship is included in the Bayesian model. Finally, a dated sample was taken from an occupation III pit (D40), which was located just north of the two preceding features and included redwares (Fig 4e).

The two other dated samples were obtained from occupations IV and III. One was located in the southeast area (D156), and the other was located in the western area (D44). D156 was a hearth located on the opposite (southern) side of the main east–west compound wall from D143 and D139 [33:Figs 3.8 and 3.14, 34:Fig 6.4 and 6.6]. As with D143 and D139, excavators identified D156 as having been produced after the construction of the original occupation IV compound walls; however, its direct stratigraphic relationship to those features in the northeastern space remains uncertain. Likewise, the midden (D44) in the western space, which excavators considered to

be an occupation III feature, has an ambiguous stratigraphic relationship with the other four features. Occupations IV and III were not always well-demarcated stratigraphically, so field reports describe an intermediate III–IV phase. For this reason, the two occupations are grouped as a single phase in the Bayesian model. The median dates from occupations III–IV span *~AD 920–1000*, and the modeled end date for these occupations, which we here consider the end of permanent residence, is *~AD 1020 (970–1110, 95%)*. This end date's probability curve extends to the right, which faithfully reflects the open possibility that residence could have extended after the median estimate for abandonment.

The final occupation (I–II) is characterized by a notably different use of space, perhaps following a brief abandonment [33:85–88]. However, this late occupation was poorly preserved, and it is possible that it appears distinct because it was located close to the contemporary surface of a field that had been plowed extensively in the twentieth century. For the Bayesian model, we treat occupation I–II as the post-abandonment phase of activity in Mollo Kontu South; however, there is not a consensus among the authors that this is so. That is, some consider the hodgepodge of features and lack of evidence for permanent occupation to be, primarily, the product of modern agriculture having obliterated these later contexts. The following provisionally disregards the possibility that modern activity destroyed the evidence for the latest permanent occupation of sector D.

The final occupation in sector D was dominated by intrusive ash pits and middens, with little standing architecture, all characteristic of late ephemeral and non-residential activity at Tiwanaku. The three most notable features of occupation I–II were a line of stones running northeast–southwest, the foundation of the intersection of two orthogonal walls, and a relatively large hearth (D36). The line of stones was unlike earlier wall foundations for two reasons: 1) it was not oriented along the Tiwanaku-period standard orientation, and 2) had a subtle curve. One possibility is that these stones are the remains of a large corral [34:170]. While the large hearth and the heavily disturbed wall foundations may indicate that there was domestic architecture constructed during this phase, the stratigraphic relationship among these features was not well defined due to the disturbed conditions of near-surface strata. The lone dated sample from occupation I–II came from the hearth (D36), *~AD 1030 (980–1150, 95%; Beta-275870)*. While a sherd from a stylistically early escudilla (Fig 4f) was found within this hearth, it is probable that it had been previously extracted from one of the many occupation I–II pits that cut into earlier strata (*pace* Augustine 2019:186).

This pattern of probable temporary occupation is echoed in sectors A and F [28]. While the late dated contexts (D36 and A111) include redwares, temporary occupants could have dug these sherds up out of earlier strata or even brought them from elsewhere.

To summarize the dates from Mollo Kontu, there are 13 dated burials and nine non-burial domestic contexts. In Mollo Kontu south, six early burials from sectors A and C have medians of ~AD 570–790; one is later at ~AD 970. Burials on the Mollo Kontu mound are all much later and can be split into two phases: four burials from ~AD 940–1000 and three from ~AD 1090–1300. As for domestic contexts, sector D's three occupations have median spans of *~AD 680–750*, *~AD 920–1000*, and *~AD 1030–1040* (two, five, and two dates, respectively). There is a notable gap in the ninth century, which follows a site-wide trend. Here, the apparent hiatus has a modeled duration of *160 years (10–280, 95%)*. However, sampling issues may mean that the duration of the hiatus is overestimated. The end of permanent residence *~AD 1020* was followed by a final occupation, mostly likely by temporary residents/visitors. The last two burial dates are among the latest at Tiwanaku.

The Putuni Complex

The labor-intensive construction and sumptuous goods found in the Putuni complex provide one of the key sets of evidence that document the long-term history of high-status residence at Tiwanaku (Fig S2.7). This sector stands out from other residential areas for its "elegant architectural elements" and higher frequencies of serving vessel sherds [35:43]. We have eight radiocarbon dates that span its entire occupation. Here, we recalibrated these dates and ordered them into a Bayesian depositional sequence, which provides much improved date estimates with lower error ranges for the complex's three stratigraphically-discrete occupations: sub-Putuni, Tiwanaku IV, and Tiwanaku V.

The sub-Putuni courtyard contexts overlay sterile soil and are dated with a single charcoal sample from the surface of a 50-cm deep red clay platform, *~AD 560* (OS-11306) [22:230, 36:61, 117, cf. 37:214, 216]. This early date agrees with indirectly associated ceramics made in transitional LF–Tiwanaku styles from the Terminal Late Formative [3, 22:229]. Structures from this initial phase were leveled and overlaid by the Early Tiwanaku occupation. This includes an artifact-rich occupation with communal kitchen areas and a system of stone drainage conduits. Two domestic use surfaces date to *~AD 730* and *~AD 820* (SMU-2369, OS-10643) [22:234, 36:62–63, 190].

There are three burials with very similar dates, *~AD 710–720*. The first is from human bone on a female who has some Amazonian and/or Chaco ancestry [38:7]; the sample was found along the exterior southern side of the courtyard, in a context disturbed by deep looters' pits (TW063, Wk-49175). The other two individuals are from an attached mortuary complex [22:238, 36:199]. The first is from an oversized urn that is over one meter in diameter and has 3-cm thick walls (feature 126, AA-107591) [36:196]. The second was a woman buried with elaborate grave goods including a beaded necklace, copper bracelets, a mirror, and a gold plate with a human face (AA-107590) [36:196, 265, Fig 6.17]. Of the five dates from the Tiwanaku IV occupation, four are very similar, *~AD 710–730*, and one is slightly later, *~AD 820*.

The adjacent Kheri Kala complex (50×75 m) was probably occupied around the same time, based on stratigraphic superpositions [22:244, 39:244]. The only date from the Kheri Kala is not included here because its context is unclear and the date falls centuries later than the expected Tiwanaku IV occupation (M-1049) [40:Table 14]. At the end of the Tiwanaku IV occupation, structures were razed and elaborate offerings were left below the subsequent construction projects [22:245–248].

The subsequent Tiwanaku V occupation completely covered the previous occupation with deep layers of clay and gravel. Over this, the 50×70 m Putuni courtyard, that we see today, was built [22:248–251]. The courtyard was lined with reused sandstone and andesite ashlars, in an "opportunistic use of geometric stones" – joints only meet at the face, hiding details that indicate they were originally made or part of other buildings such as the Pumapunku [22:248, 41:24]. Niches surround the courtyard, and the layout is identical to the inner courtyard of the earlier Kalasasaya and much older monuments at other sites in the region such as Formative period Chiripa and Pucara [43]. There was a decapitated 1.2-m extended-arm monolith in the approximate center of the courtyard [22:428, 43:30]. Stratigraphic associations date this courtyard to around the time as the attached residential patio group *~AD 910* (see below).

The residential patio follows the site-wide trends for spatial organization but architecture is more elaborate, for example, the patio is lined with flagstones. Excavators call this the "palace of the multicolored rooms" because its walls were painted in "brilliant hues of red, yellow, orange, green, and blues" [22:251]. In addition to utilitarian pottery, the occupation includes decorated Tiwanaku V vessels, jewelry, projectile points, beads, large jars for chicha, and burials with remarkable grave goods. The occupation is dated with three dates from a single tomb, which was placed after the courtyard was built. Since they are from the same depositional context, we combined in the model, which suggests the context was placed *~AD 910* (*770–1000, 95%*; Feature 18; SMU-2465, SMU-

2466, SMU-2467) [22:255]. Grave goods include a small gold pendant, a large decorated tinaja, a burnished black vessel in the shape of a llama, and ten Tiwanaku V tazones. This context was placed before the structure was abandoned [36:273], and we built this into the Bayesian model's sequence.

Finally, the end of the Putuni's occupation is dated by a sample from a collapsed roof beam. This beam was harvested *~AD 950 (780–1050, 95%,* SMU-2472) [22:251], but we do not know how much time passed between harvesting the beam and the roof's collapse. This is reflected in the wide error range for the ending boundary of the Bayesian model: *~AD 1000 (800–1210, 95%).* Excavation data are clearer on the nature of the abandonment: it was abrupt and perhaps violent, as suggested by smashed storage vessels overlying use surfaces, unprocessed carcasses, the lack of closing offerings, and the burned roof [22:257–262]. Future research could better estimate the timing of this important event by dating samples of the charred grass used in the roof. For now, we only have two dated events to estimate the timing of the monumental Putuni courtyard and the attached residential occupation. Unlike other residential sectors, there are no signs of subsequent temporary reoccupation.

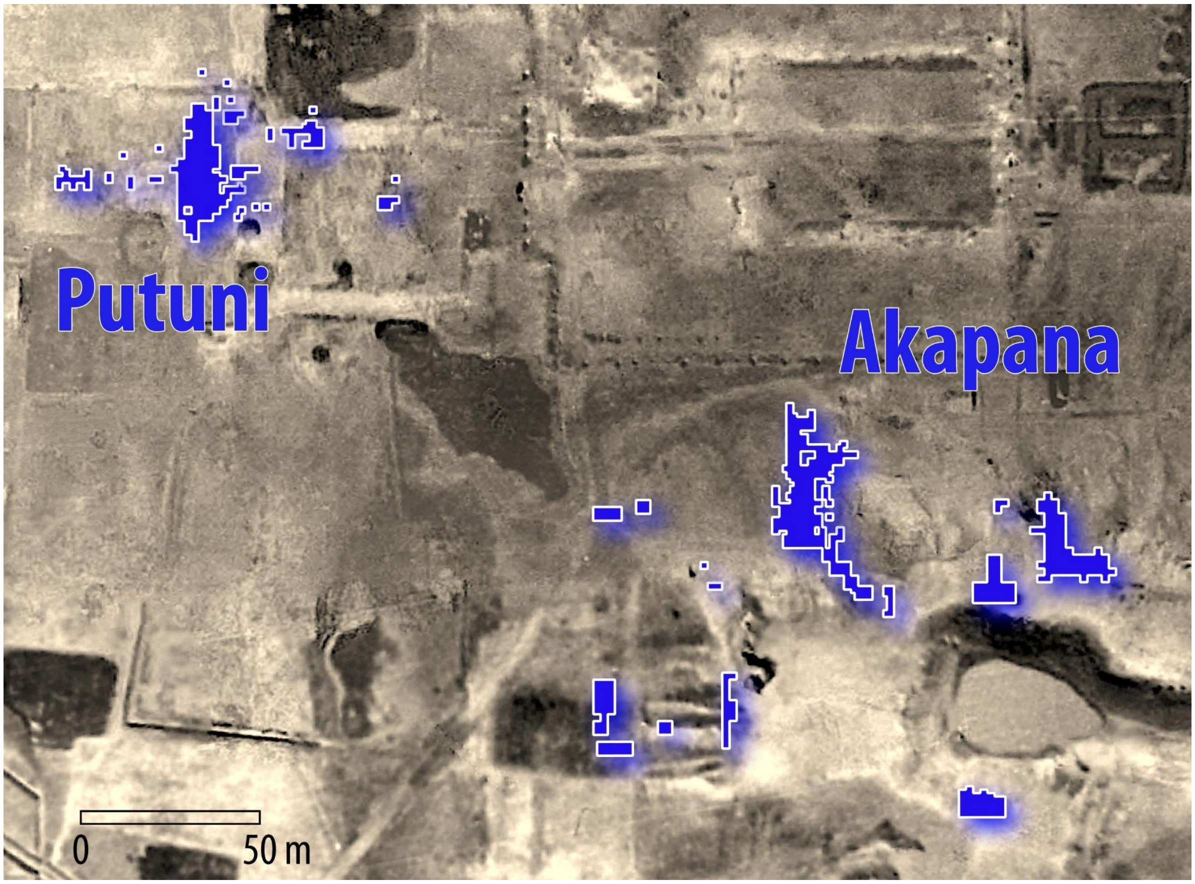

Fig S2.7. Detail of the Putuni and Akapana, showing units excavated by project Wila Jawira.

The Pumapunku

The Pumapunku was one of Tiwanaku's most important monuments and admired for its large and finely carved blocks. Here we summarize previous descriptions of the construction sequence and include new precise dates in an updated Bayesian model (Fig S2.8) [7:141, 44, 45]. The model includes all dates from the Pumapunku, even though some are before and after the Tiwanaku period (S2–S4 Files).

The Bayesian model estimates that construction began *~AD 580*, based on the starting boundary. This date agrees with early ceramics from an early offering (OS-17860) [7:133, 46]. Another early offering had been disturbed and its contents were dispersed in a level with a similar date (AA-65280). Both of these dates are grouped as a phase of initial fills, with modeled medians of *~AD 600 and 610*, from the fill below the green surface. The model interpolates this surface at *~AD 630 (590–660)*. Next, a sandy fill layer was placed. From within this fill two samples of charcoal flecks were dated from units P-11 and P-8 (UCIAMS-62884 and UCIAMS-62885, respectively). Grouped as a phase, their modeled medians are *~AD 640 and 660*. Next, a red surface was placed, interpolated at *~AD 680 (650–720, 95%).*

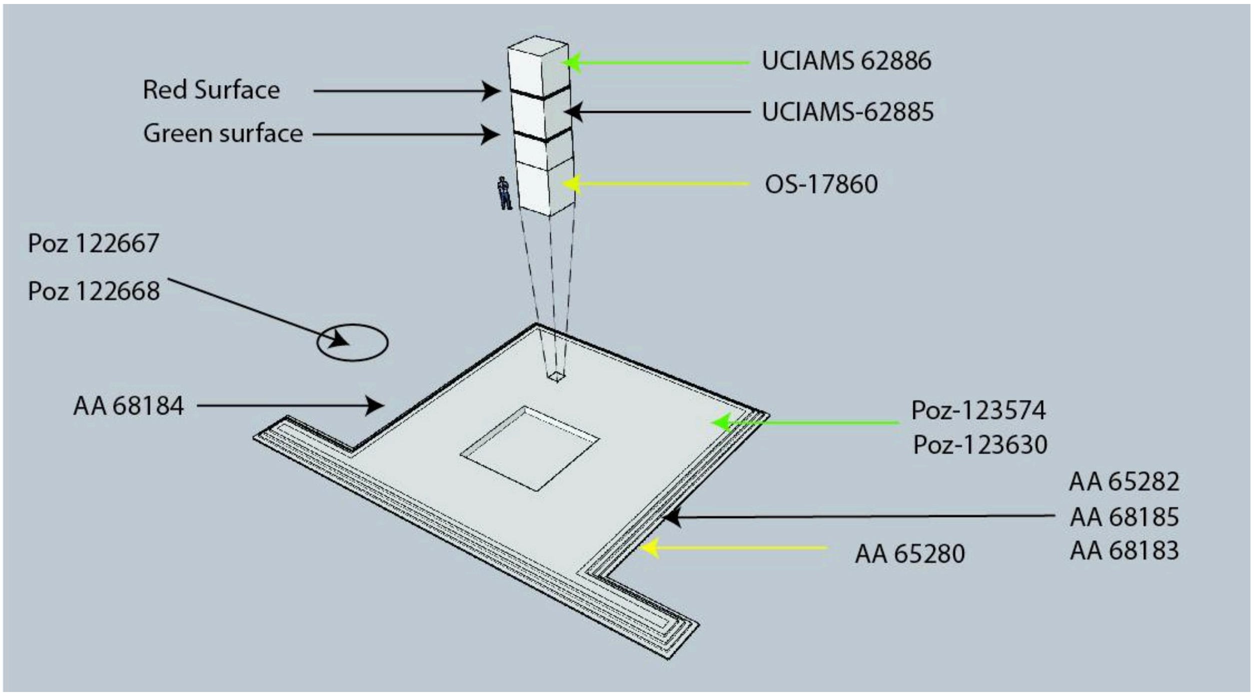

Fig S2.8. Schematic view of the Pumapunku showing the location of radiocarbon samples and the construction sequence defined by red and green surfaces.

The red surface is a clear stratigraphic component of the Pumapunku. Its date is also important because it marks the end of a masonry process that imported andesite from the distant quarries to form precise geometric blocks. From this point on, blocks are in secondary positions and construction depended on reused blocks. Hence Tiwanaku's iconic stonework of flat planes, geometric interior and exterior corners, and precision fitting front to back blocks [48] was a short affair, lasting only one generation, *~AD 590–630*, ending when the green surface was placed. The subsequent fill and red surface were contained by reused blocks, a trend that continues throughout

the site. The original plan was not completed, and all other buildings at Tiwanaku that use these impressive blocks probably pillaged them from this early phase of the Pumapunku.

Above the red surface, a final fill date helps constrain the model, *~AD 700* (UCIAMS-62886, unit P-8). Around this time or shortly after, there is a burial context (TW059). This context is from the modern surface, but it does intrude on the same construction fill as the previous date, so these dates are grouped as a phase and estimate when the final fill layer was placed. The surface context had a 10–15-year-old male [48:30]. Modeled and combined, two dates place this individual's birth around *~AD 710 (670–780, 95%; Poz-123574 & Poz-123630).* The samples are from the Petrous bone, which forms within two years of birth, so this date tells us when the individual was born. This individual's genomic sequence suggests some Amazonian and/or Chaco ancestry [38:7].

Overall, seven dates comprise a sequence of active construction at Pumapunku. In the model, First and Last queries suggest the span of construction was around *~AD 600–710.* This means that principal construction only lasted around three to four generations. Finely-cut stones were only made in the first generation, after which builders opted for reused blocks. Diagnostic ceramics from primary contexts are mostly in the earlier Tiwanaku IV style, which agrees with these construction dates [7:134].

After construction ended, earlier offerings were dug out and removed. Two of these disturbed contexts originated from below the first revetment (AA-65282 and AA-68185) and the third is from the second revetment (AA-68183; these are the group two dates in [7:Fig 11.6]). Modeled as a phase, one was around *~AD 770 (680–870, 95%; AA-68183)*; the other two are slightly earlier, *~AD 740 and ~AD 760.* These dates' probability ranges overlap and error ranges are large since they fall on a plateau on the calibration curve. A few centuries later, there was another removal of an offering along the south side of the platform *~AD 1020* (AA-68184) [7:140].

A few meters off the platform along the south side of the platform was the shallow burial of a child. Two tooth dates combine to *~AD 1090* (Individual TW100, dated by Poz-122667 & Poz-122668). The burial was found as part of a midden that included Tiwanaku period ceramics [48:36–38]. Finally, there are nine dates associated with Inca and Early Colonial material and notably, two capacocha offerings [7:136–137].

Overall, this set of dates provides us with a chronology of the Pumapunku that is coherent with dates from elsewhere at the site. Construction was a process that extended from the beginning of the Tiwanaku Period, *~AD 590–720.* The original design involved use of geometric cut stones; soon thereafter construction shifted to the use of reused stone set in an opportunistic manner. The targeted removal of offerings during the eighth century indicates a change in conceptual nature of the platform [7:134].

The Akapana

The Akapana is the largest monument at Tiwanaku (Figs 3 and S2.10). A foundation of compact clay supports this platform and seven terraces. The lower terraces consist of a double walled construction with interior retaining wall of random-coursed masonry with an exterior wall (facade) of even horizontal courses set between upright orthostats. The flat and wide summit includes structures with reused cut-stone foundations, burials, and a sunken court. The many Tiwanaku-period offerings on the Akapana show an enduring history of reverence that extends well beyond the end of permanent residential occupation and continued to the near present [50].

The first set of dates was run by the project Wila Jawira [4, 8, 16, 51]. We have corrected published errors by consulting both the original lab reports and the excavators when possible (see

notes in [38, 52]). Finally, we include unpublished and recently-published AMS dates by the University of Warsaw [39] as well as unpublished dates run by Blom and Knudson.

Most offerings are on and around the first terrace. Many of these remarkable archaeological contexts have been dated, but there are few clues to the depositional sequence. In some cases, the same architectural space was home to offerings for many centuries. This means that most dates offer only indirect clues to the chronology of construction. Here we update the depositional sequence proposed by [7:141]. These contexts are also reviewed in [38], with a focus on associated decorated ceramics.

Construction and early offerings. There are five early dates, grouped as a single phase, since these contexts were placed during the monument's principal construction. Their medians fall within a very short span, *~AD 640–660*, but error ranges are wide, ±49–211 (SMU-2329, SMU-2293, SMU-2285, ETH-6306, SMU-2468). This is contemporary with the fill at the Pumapunku between the green and red surfaces. This coincides with stylistic expectations: in the Pumapunku, this is when masons shift to reused blocks. This lines up with the beginning of construction on the Akapana, which used reused blocks from the outset.

One date is a mixture of human and animal bones dug into the Akapana's foundation, *~AD 660* (*530–810, 95%*, SMU-2329) [50:101, 53:74]. Next, on the first terrace, there was an offering of smashed ceramics, predominantly keros, between the retaining wall and the facade (Feature 2) [50:Fig 12–22, 53:94]. A second date of *~AD 640 (550–780, 95%, ETH-6306)* was taken from a cranial fragment associated with ceramics. The facade is mostly lost, but historic descriptions, maps, and photographs can be used to secure its original place and form [46, 54]. One context was dated twice (SMU-2293 and SMU-2285), allowing us to combine and model the dates to *~AD 650 (550–780, 95%).* Finally, there is a very similar date from a burial on the summit, which was placed a few meters from a large stone-lined drainage conduit *~AD 660* (*590–780, 95%*, SMU-2468) [53:44, 55:Fig 7.10]. Since it is from the construction fill of the summit, this date indicates that the moment had reached its final height by this time. This is within a century of when construction probably began, as suggested by the starting boundary of *~AD 600 (420–770, 95%).* These contexts suggest there were a few major offerings within a very short span, around the time the monument reached its full height.

Summit occupation and offering. There are four dates from the summit. The first is from the central patio of a multi roomed structure along the northern edge of the platform. The damaged form of this structure is L shaped, but the original layout may have been U shaped [46]. In the area of the central patio, there were six aligned burials that were all seated and facing north, much like a contemporary set of burials in the Putuni [36:265, 56:117]. The central figure was holding a puma-head incense burner and a bone spoon; an unpublished date calibrates to ~AD 790 (680–890, 95%; AA-107584) [53:61–62, Figs 38–40].

An elaborate offering (Feature 11) was placed in the southwest corner of the series of rooms around an open space [53:54–70]. Some camelid bones from the context overlaid the structure's foundation, indicating that it was placed after the adobe walls had fallen or were melted by rain. Bones from mostly young camelids were arranged in the room as follows: 14 camelid crania were placed face down in the north and west, eight mandibles in the southwest, long bones in the south. There was a copper pin (tupu), a copper and silver lamina, and a miniature copper sculpture of a seated fox. There was decorated and undecorated ceramics, and in the southeast, seeds from tropical plants. The arrangement reminded Manzanilla [53] of modern Aymara offerings, and Kolata [56:121] interpreted this context as a closing ceremony for the Akapana and the city of Tiwanaku. There are

two imprecise dates from this offering; combined, they place it ~AD 1000 (680–1230, 95%; SMU-2473 & INAH-972, Feature 11).

Along the north and south edges of the looter's hole the scant remains of what had been a sunken courtyard. Along the southern edge is a three-sided structure with a small set of steps that lead to a surface [53:46]. There was a small offering placed directly on this surface, which included two ceramic condor heads from incense burners and was dated to ~AD 820 (600–1030, 95%; SMU-2336). Other ceramics in the area were mostly from domestic vessels, indicating domestic activities took place, either as part of an occupation or perhaps during feast preparation [50:146, Fig 53].

Offerings above the base of the Akapana

These dates come from offerings placed in the alluvia along the base near the Akapana's northwest corner and from the first terrace. All of these dates are from offerings with human or animal bones and could have been left at any time after the Akapana's construction. It is possible that some are secondary burials.

Exterior revetments. Excavations in the 1990s dated four samples from later offerings on the second terrace and at the base of the platform. The dates have medians of ~AD 890–980 and wide error ranges. At the base of the first terrace, there was a concentration of human and llama and ceramics just north of the monument's western entrance [4, 53]. It dates to ~AD 910 (680–1040, 95%; SMU-2367; [38] incorrectly associates this date with feature 12 from a different unit, N8028 E5025 [50:101, 53:74]). Along the north side at the base of the Akapana, an incomplete carnivore (species unknown) was placed at the mouth of a conduit built into the first revetment ~AD 980 (770–1220, 95%; SMU-2330). It was reasonable to suggest that this animal was placed after the conduit ceased functioning (see photographs in [14]). [8:207, 53:83, 108, 55:186]. However, 2006 excavations showed that the conduit ends in a small room and does not connect to any conduits, so it is possible it may have never channeled water or that these blocks from a previous conduit were moved [37:219]. In either case, overlaying wind-blown deposits suggests the offering was placed shortly before a lapse with no human activity. There were no diagnostic ceramics [50:102]. Finally, two dated offerings from the second terrace have the same median: *~AD 890 (690–1030, 95% for ETH-5640 and AD 700–1020, 95% for ETH-5639).* The first includes polychrome ceramics, camelid bones, and the remains of two adolescent males with cut marks; one was found face down [17, 53] (ETH-5640, burials 1–2. This context is feature 8 from unit N8042 E5026. There is another dated feature 8 from a different unit, N8027 E5025 [16:437], since feature numbering resets for each unit. Knobloch [37:216] refers to these as features 8a and 8b, respectively). The second offering was also a young individual laid out over the much earlier ceramic smash (ETH-5639, feature 18, burial 11) [16:438–439, 37:216, 50:102, 53:74]. Based on this stratigraphic superposition, the Bayesian model assumes this context with human bone is later than the lower one. These human bones have marks from cutting and carnivores as well as exposure to the sun and wind, and were subsequently covered by wind-blown deposits. This is clear evidence that after these offerings were placed, there were no more human activities here, suggesting abandonment.

In the same part of the Akapana, there are 12 recently-processed dates on 8 individuals. Excavations in 2004 by Bolivia's *Dirección Nacional de Arqueología* found a number of closely spaced offerings along the base of Akapana's northwestern corner [57]. There are no clear stratigraphic relationships between these samples but the dates are very similar. Excavations also recovered two unusual objects, a sandstone basin and a carved stone camelid head. Five individuals were dated, four of whom were dated with duplicate radiocarbon samples (individuals TW006, 060, 065, 061, and 001). Individual TW006 is from an offering that also had animal bones, ~AD 940 (880–1000,

95%). The individual was found at the mouth of a drainage conduit, recalling the nearby carnivore offering (SMU-2330). In an adjacent unit, TW060 is from an offering with human bones, llama bones, and Tiwanaku keros and incense burners (Wk-49174) [39]. It has a very similar date, ~AD 970 (890–1020, 95%). Finally, individual TW065 was found in an offering that included 14 human crania. The date is again very similar, ~AD 940 (890–1000, 95%).

Individuals TW001 and TW061 are infants from unit 1012/2003 (which nearly aligns with N1920 E2003 on the Wila Jawira grid). This unit included nine offerings spanning a vertical depth of 46 cm (in these field reports the term *unidad estratigráfica* refers to excavation features, not units). They generally included human bones, camelid bones, and Tiwanaku redwares. Three included redwares and infant bones (features 6212, 6224, and 6225). There are two dated infants from the deepest offering, *~AD 980 (890–1030, 95%)* and *~AD 1050 (990–1160, 95%),* based on two dates each (TW001 and TW061 from feature 6225) [39]. The clear difference in dates for individuals from the same feature suggests the excavation context includes a mix of slightly older and younger material.

According to the laboratory, it is unclear whether samples TW001 and TW061 are from the same individual (processed in 2007 and 2016, respectively). In both cases, the two dates from each individual agree. However, a chi-square between the two individuals fails. This makes it most likely that these are two different infants that died at different times even though they were excavated from the same context, feature 6225. A context with mixed deposits would not be surprising, since there were few clear stratigraphic distinctions nor articulated bones to track context integrity. TW061 is one of the latest associations with Tiwanaku redwares, but the same ceramics are also associated with TW001. Hence neither date has a reliable association with these ceramics, nor have they been described in detail.

Subsequent 2009 excavations by the Tiwanaku municipality also identified human remains in the same area. This excavation was part of a conservation project that made a channel for water to drain from the top of the platform during the rainy season. All three dated individuals are from distributed contexts and no have clearly-associated grave goods [58]. Dates for the first two individuals are ~AD 1010 (900–1100, 95%; I0977) and ~AD 1100 (1020–1170, 95%; I0978). The field report mentions Tiwanaku V ceramics in a nearby context, but these do not seem to be directly associated with the human remains. A third individual was found to the east and dated to ~AD 1040 (990–1150, 95%; TW102). Another excavation from the sides of the Akapana recovered similar offerings of human bone, camelid bone, and decorated ceramics, but they have not been dated [59].

In sum, the northwestern corner of the Akapana has eight reliably-dated individuals from offerings left above the surface of the platform. Five of these individuals probably died within a half century, as suggested by medians of ~AD 940–1010. Next, there is likely a gap of a few decades before the later three individuals, ~AD 1050–1100 (I0978, TW061, TW102). The earlier burials in the sector took place during the last few generations of residential occupation at the site, while the later ones are slightly after temporary occupations. It is possible that temporary occupations continued at this time but later camps have not been dated.

North of the Akapana: group burial

About 150 m northeast of the Akapana, two nearby adjacent excavation units revealed a sequence of construction and pebbled surface with a human offering, windblown deposits, and a sacrifice pit with 16 humans, ceramics, two camelids, and bones from other animals (see photographs in [14]). There are three dates from two individuals, which can be placed in a precise depositional sequence. The first is the body of a face-down female adolescent, who was placed in the fill below a pebbled surface; the cranium protruded slightly above the surface (Feature 514) [60:231, 61:71, Figs 19–20,

62:168]. The body was placed next to a closed stone conduit, probably for water. The body is flexed, suggesting a kneeling or a fetal position, or perhaps a wrapped burial. The hands are behind her back, suggesting her hands were bound, but they are farther apart than other clearer cases of bound hands. A tooth sample dates this woman's childhood to *~AD 880 (770–970, 95%,* Wk-50229, TW097).

The second individual is from a context that is about 10 m from the first and overlays the same pebble surface. This context is an impressive set of articulated human bones from 16 humans and two camelids [62]. The humans were infants, children, and adolescents of both sexes and three have evidence for violent deaths; one of the camelids was missing its head [61:171]. A kero was directly associated with the burial, and within a few meters, 17 escudillas were found. Two teeth from individual 6, who died at age 17–20, were dated, which combine and model to *~AD 1010 (980–1140, 95%*; TW090) [62:Table 13.1]. Besides elaborate redwares (Fig 4d), there were projectile points and a clay tube (11.5×5 cm), burned on the inside, capped with a vertebra [61:67, Fig 15].

At the same depth, about 2 m to the east, there was a cache of 13 Tiwanaku IV–V escudillas [63:72], which could have been left around the same time but the context has not been dated (Fig 4d; see [61] for excavation details, ceramic analysis, and color photographs). This cache and the adjacent burials are set along the route of a shaft of sunlight that passes through the eastern gateway of the Kalasasaya during the winter solstice sunset [63:72]. This alignment may help explain the location of the burials in this area, since they are located in an open space away from buildings, which is unusual for offerings of this size and complexity. Benitez [63:73] further suggests that the number of escudillas may reference lunar months: the 12 polychrome vessels and 1 undecorated vessel, which recall 12 full lunar cycles per year in addition to 1 incomplete cycle. Crawfish remains suggest the offering was made during the rainy season.

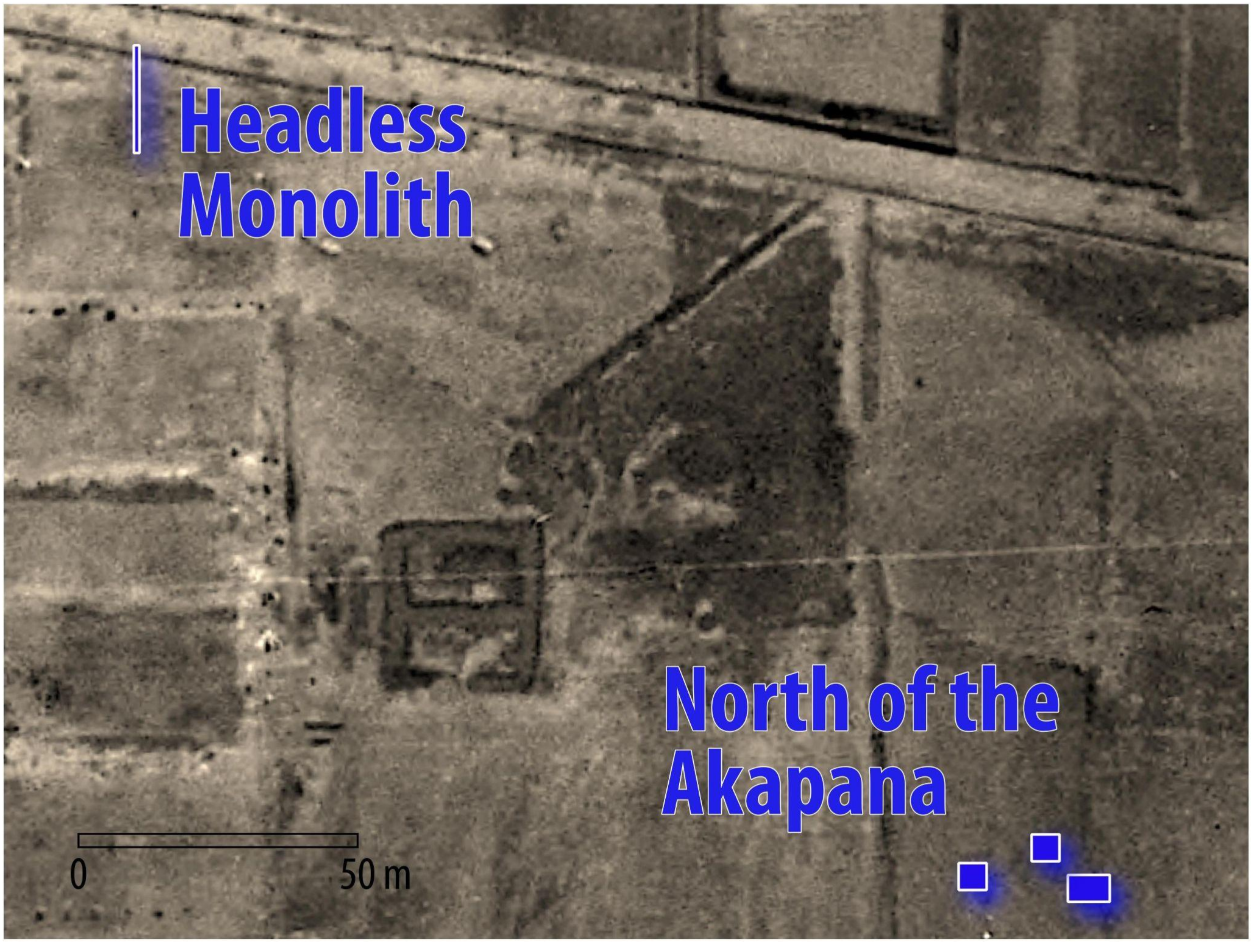

Fig S2.9. Detail of the units north of the Akapana and the trench just north of the Headless Monolith.

This pebble floor is the last dated construction project at Tiwanaku and was placed between the two dated individuals above and below the pebble surface. The Bayesian model interpolates that the pebble surface was placed *~AD 950 (780–1040, 95%)*. The floor is associated with conduits that drained the surface; using exhausted ground stone with holes in the center. Excavations west of the Akapana suggest at least four distinct pebble surfaces [34:79]. Overall, this paints the picture of an active period of community construction contemporary with the building of the Putuni platform.

North of the Kalasasaya Monument: the Headless monolith

Just north of this monolith, a 17.5 × 1 m north–south trench was excavated. There are three dates from two individuals. The Headless Monolith is located around 150 meters north of the Akapana (Fig S2.9). This sandstone monolith is similar in style and height to other Late Formative monoliths, notably Khonkho Wankane's Wila Kala [64:99]. It is unclear when it was decapitated and could have been moved from another site [65]. Material found at its base follows the pattern of an offering, left centuries after it was carved, suggesting it remained meaningful for many generations. The 2010 excavations around the monolith uncovered a dense concentration of human bone, animal bone, undecorated ceramics, and some decorated redwares, mostly in late Tiwanaku styles [66:19–23].

There were also many disarticulated human bones as well as articulated four individuals, but no formal pits or tombs. Individual 1's bones had evidence of weathering; individual 2 was buried face down. Remains of individual 3 include an articulated cranium, cervical vertebrae, and right shoulder bones, which suggest a face-down burial, possibly with the arms behind the back. Genetic analysis of this female suggests exclusively Amazonian ancestry; a tooth was dated to ~AD 930 (770–990, 95%; TW056, Wk-49173; Individual 3) [38:7]. Just below this burial was another individual with two unpublished tooth dates, which combine and calibrate to ~AD 940 (880–1000, 95%; TW064, Poz-122658 & Poz-122659; Individual 4). It was also found face down with the legs folded under the torso, perhaps kneeling, and the hands were tied behind the back.

Dates without provenience information. There are two human bone dates with scant provenience information [39]. Individual TW004 (Wk-49169) is part of a series of dates run on samples excavated by Bolivia's DINAR in 2004, mostly from the northwestern corner of the Akapana, so it could very well be from there [57]. However, the excavations did include other parts of the Akapana. TW008 (Wk-49170) is likely from a burial around three hundred meters southwest of the Akapana, recovered during museum construction [39]. These dates are very similar, with medians of ~AD 880 and ~AD 890 and the same probability range (AD 770–890, 95%). Overall, recently processed dates have very precise dates, especially on individuals with two dates. However, these data are less useful for building archaeological narratives when they are divorced from their contexts. We encourage future research to more carefully choose well-documented contexts so that radiocarbon dates and isotopes can be better integrated with archaeological data.

Site-wide model of permanent and temporary residence

For the composite model of permanent residence, we grouped dates from four sectors into early and late occupations. These are both prior to a temporal boundary between permanent and temporary residence. Here we call these phases Early, Late, and Terminal Tiwanaku, respectively. For individual sectors, there are usually too few dates to model these phases, but this can be approximated by grouping dates from all sectors. This model includes depositional sequences from individual sectors and has robust agreement indices (A_{model}=104%, $A_{overall}$=106%).

The site's initial occupation was around AD 100 during the Late Formative [1]. At this time, evidence suggests a small site with early monuments and few residents. Next, there was a surge in permanent residence in the Tiwanaku period starting around *~AD 600 (540–660, 95%; First query)*, a result that is very similar to a previous model [3:Fig 8]. In most sectors at the site, this is the earliest occupation. In all four dated residential areas, there was a clear stratigraphic boundary between early and late Tiwanaku occupations. Late occupations were often built after the area had been razed as part of urban renewal, following the pattern first documented in Akapana East [9:138]. While some models assume the site was continually occupied, these Bayesian models follow stratigraphic patterns, which both suggest a lapse in permanent residence in multiple parts of the sites. In all sectors, there are very few dates in the late AD 700s and early AD 800s, and it is possible that there were few permanent residents at this time. The combined residential model suggests that early Tiwanaku occupations ended *~AD 790 (740–880, 95%; Last query)* and late Tiwanaku occupation started *~AD 830 (770–910, 95%, First query).* The most likely gap is about a generation, but these probability ranges overlap significantly, meaning there might not have been a gap at all; this period may require additional samples. Future research will have to deal with unusual wiggles in the calibration curves around this time. Furthermore, the latest occupations are best sampled for taphonomic reasons – they are closest to the surface. Earlier occupations were likely disturbed, in addition to being less accessible to excavation.

Model for monument building

This Bayesian model groups seven dates from Pumapunku and five from the Akapana that are directly related to its construction. Run separately, these models strongly agree. The combined model maintains the same stratigraphic priors. For the Akapana, the only prior is to combine SMU-2285 & SMU-2293, since they are from the same context. For the Pumapunku, we use the same sequence: two dates, the green surface, two dates, the red surface, and finally, two more dates (see above). We combine these dates into a single phase, since the individual models are very similar. Only with additional dates can we adequately test the possibility that the construction of these and other monuments was happening at the same time, since there is no stratigraphic connection. Assuming construction was around the same time, this model provides good estimates for when the main time of Tiwanaku-period monument construction started and stopped: *~AD 590–720*.

For the AD 900s, there are only three dates. One is the pebble surface north of the Akapana, which is an interpolated date based on the stratigraphic sequence of TW090 and TW097 (see above). We also include two contexts from the Putuni. Feature 18 is dated with a combination of three dates and is stratigraphically associated with the courtyard. Similarly, the harvest date of the collapsed roof beam also helps date the construction of this part of the Putuni. This small sample of three events is the basis for a preliminary KDE (Fig 7), but overall, we have very little information on community construction projects at this time. Similar to other models, there is a noticeable lack of dates in the AD 800s.

Model for making and discarding redwares

This model includes all dates that are associated with redwares, but we do not track distinctions between the styles Tiwanaku IV and V (S1 File). For dates from residential contexts, we used posterior probability density functions exported from the models of site-wide residential contexts and monument construction, which both include sequences from the sector-specific models. This approach is more consistent with the goal of tracking site-wide patterns. We did try an alternative model that used priors from models of individual residential sectors, but the differences were negligible. This model has the most dates, 51. The strong agreement indices and precision boundaries indicate that they are a temporally coherent set of dates. This larger sample means this model is less sensitive to adding or removing dates, for example, if we mistakenly included a date not associated with redwares.

The KDE for the redwares model peaks after AD 900, but it is not clear if this reflects an increasing intensity of ceramic traditions at this time or if reflects a sample bias toward more easily excavated contexts that are closer to the surface. The boundaries of this model are also useful for tracking patterns at nearby sites. We can expect most surface scatters of redwares in the region to fall between the model boundaries, *~AD 620–1040*. However, this is not generalizable to all Tiwanaku sites. One notable exception is the Katari Valley; residents at Lukurmata and Tiraska used redwares for at least another century [4:Table 3.1, 67].

Models for placing bodies

For these models, we built single-phase models. The first includes dates from formal tombs (see S1). The second includes those with apparent violent deaths, based on seven dated individuals and contexts, which are all associated with burials of people who died violently. A few were found

face down, which is less compelling evidence for a violent death, while most are much clearer, with cut marks on fresh bone and unhealed puncture wounds.

North of the Kalasasaya at the base of the 'headless' monolith, bones from four individuals were found in a disturbed area with no formal burial pits or architecture. Bone articulations suggest they were buried with their hand behind their back or face down. Two dated individuals are included here (TW056 and TW064).

Near the mound in Mollo Kontu, some people were buried face down or kneeling, including a one dated young adult male (MK-39787 in feature 19). He had been placed "kneeling face down with his legs folded up on both sides and … his hands behind his back" [25:221–223, Fig 8.37]. He was found with five other people; perhaps they all died around the same time.

North of the Akapana, a large set of humans and camelids suggest a single sacrifice event. One individual was buried face down with her hands behind her back (TW097); another was found associated with 16 humans and two camelids (TW097). Of these, three have unhealed blunt trauma and one has multiple penetrating wounds to the head (Fig 3d–f) [61:Figs 53–55, 62:175, 179, Fig 13.17].

On the Akapana, there are two dates associated with two adolescent males with cut marks on fresh bone; one was found face down (ETH-5639, ETH-5640; burials 1–2) [16:437–439, 53:74, 218]. One date was from a 22–30-year-old (N8042 E5026, level 2a, feature 8, specimen 3188, ETH-5640). The bones have marks from cutting and carnivores as well as exposure to the sun and wind, consistent with site abandonment after they died.

So far, this is a small set of dates, but other violent deaths remain to be dated, for example, one at Muru Ut Pata. Based on the nearby radiocarbon dates, this burial probably also falls in the same lapse as the other dated violent deaths. Future work could target high-precision dating for these intriguing contexts (for example, modeling multiple dates, as we do for the Ch'iji Jawira burial).

Finally, the last model includes dates from all other contexts with human remains, excluding those in the phases for tombs and violent deaths. There are many other unpublished concentrations of human bones near the surface, which are probably part of this site-wide pattern, for example, in 2007–2008 excavations in the sector La Karaña [66:45–46]. Most likely, these were left during temporary visits after permanent occupation had ended. There are few dates, but they extend for centuries after the city's collapse.

The OxCal code (S2 File) also includes a model that includes all human remains, but we found this to be less informative so we excluded it from the main text. The earliest contexts are from tombs, similar to early permanent residence, since tombs are always found in residential compounds. Both are slightly earlier than the starting boundary for redwares, hinting that people moved here first and then began producing redwares.

For this paper, we disregarded excavators' label of some contexts as offerings, since this is a subjective category. However, we note that many apparent offerings include the pattern of animal bone, human bone, and redwares, so all three of these categories are listed in (S1 Table). Future research may be able to track temporal patterns among deposits that include all three, or apply a more formal definition of offering.

Table S2.1. Summary results from Bayesian models' boundaries and queries. The 95% error span is a useful indicator of precision. Events with larger error spans would benefit most from additional dates or modeled priors. The paper's main results are in bold, which mark the end of sustained community practices.

		**Modeled result (cal AD)**				
**Sector or Model**	**Modeled boundary or query**	**Median**	**95%**			**Error span (95%)**
Akapana East	Start	570	370	–	750	380
Akapana East	End Permanent Residence	1020	910	–	1140	230
Akapana East	End Temporary Residence	1240	990	–	1710	720
Muru Ut Pata	Start	900	710	–	1000	290
Muru Ut Pata	End	970	890	–	1150	260
Mollo Kontu, Sector D	Start	640	480	–	770	290
Mollo Kontu, Sector D	End Permanent Residence	1020	970	–	1120	150
Putuni	First (after initial Putuni occupation)	670	570	–	750	180
Putuni	End	1000	800	–	1220	420
All residential sectors	First (after initial Putuni occupation)	600	540	–	670	130
**All residential sectors**	**End Permanent & Start Temporary Residence**	**1010**	**970**	**–**	**1050**	**80**
**All residential sectors**	**End Temporary Residence**	**1050**	**990**	**–**	**1260**	**270**
Pumapunku	Start	580	500	–	640	140
Pumapunku	Last	710	680	–	780	140
Akapana	Start	600	320	–	760	440
Akapana	End	690	590	–	960	370
Monument construction	Start	590	530	–	640	110
**Monument construction**	**End (excluding tenth century contexts)**	**720**	**670**	**–**	**790**	**120**
Site-wide redwares	Start	620	570	–	650	80
**Site-wide redwares**	**End**	**1040**	**1010**	**–**	**1080**	**70**
All contexts with human bone	Start	570	510	–	600	90
All contexts with human bone	End	1300	1270	–	1360	90
Tombs	Start	560	460	–	600	140
**Tombs**	**End**	**910**	**780**	**–**	**1010**	**230**
**Violent deaths**	**Start**	**910**	**800**	**–**	**970**	**170**
**Violent deaths**	**End**	**1020**	**980**	**–**	**1090**	**110**
Neither tombs nor violent deaths (human bone)	Start	610	510	–	670	160
Neither tombs nor violent deaths (human bone)	End	1310	1270	–	1410	140

References

1. Marsh EJ. A Bayesian Re-Assessment of the Earliest Radiocarbon Dates from Tiwanaku, Bolivia. Radiocarbon. 2012;54: 203–218. doi:10.2458/azu_js_rc.v54i2.15826
2. Grupo RPP. 12 fotos del revelador hallazgo que abre una nueva página en la historia de la cultura Tiahuanaco. RPP. 25 Sep 2019. Available: https://rpp.pe/mundo/latinoamerica/bolivia-tiahuanaco-el-revelador-hallazgo-que-abre-una-nueva-pagina-en-la-historia-de-la-cultura-fotos-noticia-1221335. Accessed 13 Mar 2023.
3. Marsh EJ, Roddick AP, Bruno MC, Smith SC, Janusek JW, Hastorf CA. Temporal Inflection Points in Decorated Pottery: A Bayesian Refinement of the Late Formative Chronology in the Southern Lake Titicaca Basin, Bolivia. Latin American Antiquity. 2019;30: 798–817. doi:10.1017/laq.2019.73
4. Janusek JW. Vessels, Time, and Society: Toward a Chronology of Ceramic Style in the Tiwanaku Heartland. In: Kolata AL, editor. Tiwanaku and Its Hinterland: Archaeological and Paleoecological Investigations of an Andean Civilization, Vol 2: Urban and Rural Archaeology. Washington, D.C.: Smithsonian Institution Press; 2003. pp. 30–92.
5. Vranich A. Interpreting the Meaning of Ritual Spaces: The Temple Complex of Pumapunku, Tiwanaku, Bolivia. PhD Dissertation, Department of Anthropology, University of Pennsylvania. 1999.
6. Yaeger J, López Bejarano JM. Reconfiguración de un espacio sagrado los inkas y la pirámide “Pumapunku” en Tiwanaku, Bolivia. Chungara. 2004;36: 337–350. doi:10.4067/S0717-73562004000200008
7. Yaeger J, Vranich A. A Radiocarbon Chronology of the Pumapunku Complex and a Reassessment of the Development of Tiwanaku, Bolivia. In: Vranich A, Levine A, editors. Advances in Titicaca Basin Archaeology–2. Los Angeles: Cotsen Institute of Archaeology, University of California; 2013. pp. 127–146.
8. Janusek JW. Identity and Power in the Ancient Andes: Tiwanaku Cities through Time. New York: Routledge; 2004.
9. Janusek JW. State and Local Power in a Prehispanic Andean Polity: Changing Patterns of Urban Residence in Tiwanaku and Lukurmata, Bolivia. PhD Dissertation, Department of Anthropology, University of Chicago. 1994.
10. Janusek JW. The Changing Face of Tiwanaku Residential Life: State and Social Identity in an Andean City. In: Kolata AL, editor. Tiwanaku and Its Hinterland: Archaeological and Paleoecological Investigations of an Andean Civilization, Vol 2: Urban and Rural Archaeology. Washington, D.C.: Smithsonian Institution Press; 2003. pp. 264–295.
11. Janusek JW. Residential Diversity and the Rise of Complexity in Tiwanaku. In: Stanish C, Cohen AB, Aldenderfer MS, editors. Advances in Titicaca Basin Archaeology-1. Los Angeles: Cotsen Institute of Archaeology, University of California; 2005. pp. 143–171.
12. Janusek JW. Residence and Ritual in Tiwanaku: Hierarchy, Specialization, Ethnicity, and Ceremony. In: Manzanilla LR, Chapdelaine C, editors. Domestic Life in Prehispanic Capitals: A Study of Specialization, Hierarchy, and Ethnicity. Ann Arbor: Museum of Anthropology, University of Michigan; 2009. pp. 159–179.
13. Vella M-A, Ernenwein EG, Janusek JW, Koons M, Thiesson J, Sanchez C, et al. New insights into prehispanic urban organization at Tiwanaku (NE Bolivia): Cross combined approach of photogrammetry, magnetic surveys and previous archaeological excavations. Journal of Archaeological Science: Reports. 2019;23: 464–477. doi:10.1016/j.jasrep.2018.09.023
14. Kolata AL. Online Tiwanaku, a Digital Companion to: Kolata AL, editor. Tiwanaku Its

Heartland Hinterland, Vols. 1 & 2. Washington, D.C.: Smithsonian Institution Press; 2003. University of Chicago: OCHRE Data Service; 2019. Available: https://ochre.lib.uchicago.edu/PWJ/
15. Janusek JW. Collapse as Cultural Revolution: Power and Identity in the Tiwanaku to Pacajes Transition. Archaeological Papers of the American Anthropological Association. 2004;14: 175–209. doi:https://doi.org/10.1525/ap3a.2004.14.175
16. Alconini Mujica S. Rito, símbolo e historia en la pirámide de Akapana, Tiwanaku: un análisis de cerámica ceremonial prehispánica. La Paz, Bolivia: Acción; 1995.
17. Blom DE, Janusek JW, Buikstra JE. A Reevaluation of Human Remains from Tiwanaku. In: Kolata AL, editor. Tiwanaku and Its Hinterland: Archaeological and Paleoecological Investigations of an Andean Civilization, Vol 2: Urban and Rural Archaeology. Washington, D.C.: Smithsonian Institution Press; 2003. pp. 435–446.
18. Rivera Casanovas C. Ch'iji Jawira: A Case of Ceramic Specialization in the Tiwanaku Urban Periphery. In: Kolata AL, editor. Tiwanaku and Its Hinterland: Archaeological and Paleoecological Investigations of an Andean Civilization, Vol 2: Urban and Rural Archaeology. Washington, D.C.: Smithsonian Institution Press; 2003. pp. 296–315.
19. Becker SK. Skeletal evidence of craft production from the Ch'iji Jawira site in Tiwanaku, Bolivia. Journal of Archaeological Science: Reports. 2016;9: 405–415. doi:10.1016/j.jasrep.2016.08.017
20. Millard AR, Annis RG, Caffell AC, Dodd LL, Fischer R, Gerrard CM, et al. Scottish soldiers from the Battle of Dunbar 1650: A prosopographical approach to a skeletal assemblage. PLOS ONE. 2020;15: e0243369. doi:10.1371/journal.pone.0243369
21. AlQahtani SJ, Hector MP, Liversidge HM. Brief communication: The London atlas of human tooth development and eruption. American Journal of Physical Anthropology. 2010;142: 481–490. doi:10.1002/ajpa.21258
22. Berryman CA. Food, Feasts, and the Construction of Identity and Power in Ancient Tiwanaku: A Bioarchaeological Perspective. PhD Dissertation, Department of Anthropology, Vanderbilt University. 2010. Available: https://ir.vanderbilt.edu/handle/1803/10780
23. Couture NC, Sampeck K. Putuni: A History of Palace Architecture at Tiwanaku. In: Kolata AL, editor. Tiwanaku and Its Hinterland: Archaeological and Paleoecological Investigations of an Andean Civilization, Vol 2: Urban and Rural Archaeology. Washington, D.C.: Smithsonian Institution Press; 2003. pp. 226–263.
24. Roddick AP, Cuynet F. Genealogies and Juxtapositions: Traces of Potting Communities and Firing Facilities in Lake Titicaca Basin. J Archaeol Method Theory. 2020; 1143–1171. doi:10.1007/s10816-020-09491-6
25. Ponce Sanginés C. Informe de labores (octubre 1957 - febrero 1960). La Paz, Bolivia: Centro de Investigaciones Arqueológicas en Tiwanaku; 1961. Unpublished report available upon request to the corresponding author.
26. Couture NC. Ritual, Monumentalism, and Residence at Mollo Kontu, Tiwanaku. In: Kolata AL, editor. Tiwanaku and Its Hinterland: Archaeological and Paleoecological Investigations of an Andean Civilization, Vol 2: Urban and Rural Archaeology. Washington, D.C.: Smithsonian Institution Press; 2003. pp. 202–225.
27. Rodas D, Fontenla R, Arraita E. Excavaciones en el Montículo de Mollo Kontu (Sector MK-M). In: Couture NC, Blom DE, Bruno MC, editors. Proyecto Arqueológico Jach'a Marka: Informe de Investigaciones Realizadas en 2007. La Paz: La Unidad Nacional de Arqueología; 2007. pp. 31–48. Available: https://osf.io/z2qhv/
28. Fontenla R, Ulloa D. Excavaciones de Area F. In: Couture NC, Blom DE, Bruno MC, editors. Proyecto Arqueológico Jach'a Marka: Informe de Investigaciones Realizadas en 2008. La Paz:

La Unidad Nacional de Arqueología; 2010. pp. 47–67. Available: https://osf.io/z2qhv/
29. Couture NC, Blom DE, Bruno MC. Proyecto Arqueológico Jach'a Marka: Informe de Investigaciones Realizadas en 2006. La Paz: La Unidad Nacional de Arqueología; 2006. Available: https://osf.io/z2qhv/
30. Couture NC, Blom DE, Bruno MC. Proyecto Arqueológico Jach'a Marka: Informe de Investigaciones Realizadas en 2007. La Paz: La Unidad Nacional de Arqueología; 2007. Available: https://osf.io/z2qhv/
31. Couture NC, Blom DE, Bruno MC. Proyecto Arqueológico Jach'a Marka: Informe de Investigaciones Realizadas en 2008. La Paz: La Unidad Nacional de Arqueología; 2010. Available: https://osf.io/z2qhv/
32. Blom DE, Couture N. Diversidad urbana en Tiwanaku: arqueología funeraria y residencial en Mollo Kontu. Proyecto arqueológico Jach'a Marka, temporada 2001. La Paz: Direccion Nacional de Arqueología; 2004.
33. Rodas D. Proyecto Arqueológico Jach'a Marka: Informe de Investigaciones Realizadas en 2006. In: Couture NC, Blom DE, Bruno MC, editors. La Paz: La Unidad Nacional de Arqueología; 2006. pp. 2–51. Available: https://osf.io/z2qhv/
34. Mattox CW. Materializing Value: A Comparative Analysis of Status and Distinction in Urban Tiwanaku, Bolivia. Master's Thesis, McGill University. 2011.
35. Augustine JMF. Style, Aesthetics, and Politics: Polychrome Ceramic Iconography in the Tiwanaku Valley, AD 500-1100. Ph.D., The University of Chicago. 2019. Available: http://search.proquest.com/docview/2311072388/abstract/839A4A82457B423FPQ/1
36. Janusek JW. Out of Many, One: Style and Social Boundaries in Tiwanaku. Latin American Antiquity. 2002;13: 35–61.
37. Couture NC. The Construction of Power: Monumental Space and Elite Residence at Tiwanaku, Bolivia. PhD Dissertation, Department of Anthropology, University of Chicago. 2002.
38. Knobloch PJ. Tiwanaku's Coming of Age: Refining Time and Style in the Altiplano. In: Vranich A, Stanish C, editors. Visions of Tiwanaku. Los Angeles: Cotsen Institute of Archaeology, University of California; 2013. pp. 211–233.
39. Popović D, Molak M, Ziółkowski M, Vranich A, Sobczyk M, Vidaurre DU, et al. Ancient genomes reveal long-range influence of the pre-Columbian culture and site of Tiwanaku. Sci Adv. 2021;7. doi:10.1126/sciadv.abg7261
40. Escalante Moscoso JF. Arquitectura prehispánica en los Andes bolivianos. La Paz, Bolivia: CIMA; 1994.
41. Ponce Sanginés C. Tiwanaku: espacio, tiempo y cultura. La Paz, Bolivia: Editorial Los Amigos del Libro; 1981.
42. Vranich A. The Development of the Ritual Core of Tiwanaku. In: Young-Sánchez M, editor. Tiwanaku: Papers from the 2005 Mayer Center Symposium at The Denver Art Museum. Denver: Denver Art Museum; 2009. pp. 11–34.
43. Mohr-Chávez KL. The Significance of Chiripa in Lake Titicaca Basin Developments. Expedition. 1988;30: 17–26.
44. Guengerich A, Janusek JW. The Suñawa Monolith and a Genre of Extended-Arm Sculptures at Tiwanaku, Bolivia. Ñawpa Pacha. 2021;41: 19–46. doi:10.1080/00776297.2020.1830974
45. Vranich A. The Construction and Reconstruction of Ritual Space at Tiwanaku, Bolivia. Journal of Field Archaeology. 2006;31: 121–136. doi:10.1179/009346906791071990
46. Vranich A. Continuity and Transformation in Tiwanaku Ritual Architecture. Peruvian Archaeology. 2020;4: 31–81.
47. Cordero Miranda G. Informe preliminar acerca de las excavaciones en Pumapunku. La Paz:

Unidad Nacional de Arqueología; 1978. Unpublished report available upon request to the corresponding author.
48. Protzen J-P, Nair S. The stones of Tiahuanaco: a study of architecture and construction. Los Angeles: Cotsen Institute of Archaeology Press, University of California, Los Angeles; 2013.
49. Hidalgo Rocabado R, Alvarez Mamani C, Arratia Velasco E, Ramos Fernández M, Ticona Choque C. Proyecto de conservación de la pirámide de Puma Punku, sector sur 2012. Municipalidad de Tiwanaku; 2012. Unpublished report available upon request to the corresponding author.
50. Bandelier AF. The Ruins at Tiahuanaco. Proceedings of the American Antiquarian Society. 1911;21: 218–265.
51. Kolata AL, editor. Tiwanaku and Its Hinterland: Archaeological and Paleoecological Investigations of an Andean Civilization, Vol. 2: Urban and Rural Archaeology. Washington, D.C.: Smithsonian Institution Press; 2003.
52. Owen B. Distant Colonies and Explosive Collapse: The Two Stages of the Tiwanaku Diaspora in the Osmore Drainage. Latin American Antiquity. 2005;16: 45–81. doi:10.2307/30042486
53. Manzanilla L. Akapana: una pirámide en el centro del mundo. México, D.F.: Instituto de Investigaciones Antropológicas, Universidad Nacional Autónoma de México; 1992.
54. Vranich A. La Pirámide de Akapana: Reconsiderando el Centro Monumental de Tiwanaku. Kaulicke P, Isbell WH, editors. Boletín de Arqueología PUCP. 2001;5: 295–308. doi:10.18800/boletindearqueologiapucp.200101.011
55. Kolata AL. Tiwanaku Ceremonial Architecture and Urban Organization. In: Kolata AL, editor. Tiwanaku and Its Hinterland: Archaeological and Paleoecological Investigations of an Andean Civilization, Vol 2: Urban and Rural Archaeology. Washington, D.C.: Smithsonian Institution Press; 2003. pp. 175–201.
56. Kolata AL. The Tiwanaku: portrait of an Andean civilization. Cambridge, MA: Blackwell; 1993.
57. Escalante Moscoso JF, editor. Informe de la excavación arqueológica de la pirámide de Akapana. La Paz: Dirección Nacional de Arqueología; 2004. Unpublished report available upon request to the corresponding author.
58. Nakatsuka N, Lazaridis I, Barbieri C, Skoglund P, Rohland N, Mallick S, et al. A Paleogenomic Reconstruction of the Deep Population History of the Andes. Cell. 2020;181: 1–15. doi:10.1016/j.cell.2020.04.015
59. Bejar Luksic LF. Report of archaeological excavation in Akapana, Tiwanaku, Bolivia, season 2006. Tiwanaku: Akapana Archaeological Project (PAAK), Dirección Nacional de Arqueología; 2022.
60. Koons ML. Architectural and Sociopolitical Organization: Integrating Ground-Penetrating Radar and Archaeological Excavations at Tiwanaku, Bolivia. Master's Thesis, Department of Anthropology. 2006.
61. Rivera AF. Espacios ceremoniales al pie de Akapana: excavaciones de las unidades N2043-E1023 / N2043-E1024. Licenciatura Thesis, Pontificia Universidad Católica del Perú. 2011.
62. Verano JW. Excavation and Analysis of Human Skeletal Remains from a New Dedicatory Offering at Tiwanaku. In: Vranich A, Levine A, editors. Advances in Titicaca Basin Archaeology–2. Los Angeles: Cotsen Institute of Archaeology, University of California; 2013. pp. 167–180.
63. Benitez L. Descendants of the Sun: Calendars, Myth, and the Tiwanaku State. In: Young-Sánchez M, editor. Tiwanaku: Papers from the 2005 Mayer Center Symposium at The Denver Art Museum. Denver: Denver Art Museum; 2009. pp. 49–82.
64. Janusek JW, Ohnstad AT. Stone stelae of the southern basin: a stylistic chronology of ancestral personages. In: Isbell H, Uribe M, Tiballi A, Zegarra EP, editors. Images in Action: The

Southern Andean Iconographic Series. Los Angeles: Cotsen Institute of Archaeology Press; 2018. pp. 79–106. Available: https://dig.ucla.edu/sais/images-in-action-visual-database?field_chapter_target_id=217

65. Roddick AP, Janusek J. Moving between Homes Landscape, Mobility, and Political Action in the Titicaca Basin. In: Jennings J, Swenson E, editors. Powerful Places in the Ancient Andes. Albuquerque: University of New Mexico Press; 2018. pp. 287–322. Available: https://muse.jhu.edu/book/60581
66. Jiménez Balderrama V. Proyecto de conservación monolito descabezado. Bolivia: Gobierno municipal de Tiwanaku; 2010. Unpublished report available upon request to the corresponding author.
67. Korpisaari A. Death in the Bolivian High Plateau: Burials and Tiwanaku Society. Oxford: British Archaeological Reports; 2006.
