# Supplementary material for: The center cannot hold: A Bayesian chronology for the collapse of Tiwanaku
Source: PLoS One. 2023 Nov 22;18(11):e0288798. doi: 10.1371/journal.pone.0288798 (PMC10664893; doi:10.1371/journal.pone.0288798)
Supplement: S3 File — (PDF) [file pone.0288798.s004.pdf]

Supplementary file for:

Marsh EJ, Vranich A, Blom D, Bruno M, Davis K, Augustine J, et al. The center cannot hold: A Bayesian chronology for the collapse of Tiwanaku. PLOS ONE. 2023;18: e0288798. doi:10.1371/journal.pone.0288798

S3. OxCal code

Late Formative dates

```
 Plot()
 {
  Curve("IntCal20","IntCal20.14c");
  Curve("SHCal20","SHCal20.14c");
  Mix_Curves("Tiwanaku
mix","SHCal20","IntCal20",P(-1,101,[0,1,1,1,1,1,1,1,1,1,1,0,0,0,0,0,0,0,0,0,0,8,8,8,8,8,8
,8,8,8,20,20,20,20,20,20,20,20,20,20,20,20,20,20,20,20,20,20,20,20,11,11,11,11,11,11,1
1,11,11,11,9,9,9,9,9,9,9,9,9,9,2,2,2,2,2,2,2,2,2,2,0,0,0,0,0,0,0,0,0,0,0,0,0,0,0,0,0,0,0,0,0
]));
  Sequence()
  {
   Boundary("Start Late Formative Tiwanaku");
   KDE_Plot()
   {
    R_Date("Kk'arana 1.5.4",1742,49);
    R_Date("Kk'arana 1.19.12",1798,49);
    R_Date("Kk'arana 1.12.8.B",1753,50);
    Sequence("Bennett V and Kidder A")
    {
     Date("Bennett V Level 9");
     R_Date("Bennett V Level 8, 3.5-4.0 m, Kidder A Level 15, 3.5–3.75 m",1817,103);
     Date ("Bennett V Level 7-8, Clear stratigraphic break");
     R_Date("Bennett V Level 7, 3.0–3.5 m, Kidder A Level 14, 3.25–3.5 m",1692,104);
     R_Date("Bennett V Level 6, 2.5–3.0 m, Kidder A Level 12, 2.75–3.0 m",1707,93);
     R_Date("Bennett V Level 5. 2.0–2.5 m, Kidder A Level 9, 2.0–2.25 m",1702,103);
    };
    Sequence("Sub-Kalasasaya")
    {
     Phase("Stratum 7")
     {
      Sequence("D-14")
      {
       R_Date("GaK-193",1850,90);
       R_Date("GaK-192",1990,100);
```

```
      };
      R_Date("P-532a",1956,76) & R_Date("P-532b",1847,75);
      R_Date("M-1051",1780,150);
      R_Date("M-1050",1760,150);
     };
     Phase("Stratum 5")
     {
      R_Date("P-534",1866,62);
      R_Date("GaK-195",1750,100);
      R_Date("Hv-19",1645,80);
     };
    };
   };
   Boundary("End Late Formative Tiwanaku");
  };
 };
```

Akapana East

```
Plot()
 {
  Curve("IntCal20","IntCal20.14c");
  Curve("SHCal20","SHCal20.14c");
  Mix_Curves("Tiwanaku
mix","SHCal20","IntCal20",P(-1,101,[0,1,1,1,1,1,1,1,1,1,1,0,0,0,0,0,0,0,0,0,0,8,8,8,8,8,8,8
,8,8,8,20,20,20,20,20,20,20,20,20,20,20,20,20,20,20,20,20,20,20,20,11,11,11,11,11,11,1
1,11,11,11,9,9,9,9,9,9,9,9,9,9,2,2,2,2,2,2,2,2,2,2,0,0,0,0,0,0,0,0,0,0,0,0,0,0,0,0,0,0,0,0
]));
  Sequence()
  {
   Boundary("Start");
   KDE_Plot("Tiwanaku period dates")
   {
    R_Date("AKE1, AA-107585, cyst burial",1335,26);
    R_Date("AKE2, AA-107586, three-chambered burial",1209,25);
    Sequence("Three phases of occupation")
    {
     KDE_Plot("First occupation phase")
     {
      R_Date("AKE1M, SMU-2333",1716,276);
      R_Date("AKE1M, Beta-55491",1500,100);
      R_Date("AKE1, SMU-2471",1428,99);
     };
     KDE_Plot("Middle phase")
     {
```

```
     First("First date for middle phase");
     Last("Last date for middle phase");
     R_Date("AKE1, SMU-2469",1191,101);
     R_Date("AKE1, SMU-2277",1134,60);
     R_Date("AKE1, SMU-2278",1246,112);
     R_Date("AKE1, SMU-2289",1185,56);
     R_Date("AKE1, SMU-2276",1073,57);
     R_Date("AKE1, SMU-2290",1121,74);
    };
   };
  };
  Boundary("End of permanent residence");
  KDE_Plot("Final phase of temporary occupation")
  {
   R_Date("AKE1M, SMU-2332",1017,115);
   R_Date("AKE1M, SMU-2331",872,197);
   R_Date("AKE1M, SMU-2740",632,183);
  };
  Boundary("End");
 };
};
```

Muru Ut Pata

```
 Plot()
 {
  Curve("IntCal20","IntCal20.14c");
  Curve("SHCal20","SHCal20.14c");
  Mix_Curves("Tiwanaku
mix","SHCal20","IntCal20",P(-1,101,[0,1,1,1,1,1,1,1,1,1,1,0,0,0,0,0,0,0,0,0,0,8,8,8,8,8,8,8
,8,8,8,20,20,20,20,20,20,20,20,20,20,20,20,20,20,20,20,20,20,20,20,11,11,11,11,11,11,1
1,11,11,11,9,9,9,9,9,9,9,9,9,9,2,2,2,2,2,2,2,2,2,2,0,0,0,0,0,0,0,0,0,0,0,0,0,0,0,0,0,0,0,0,0
]));
  Sequence()
  {
   Boundary("Start Muru Ut Pata");
   KDE_Plot("Main occupation")
   {
    R_Date("UGAMS-26434, R-13-8, principal occupation",1110,20);
    R_Date("UGAMS-26432, R-10-14, shaft tomb, individual 11",1120,20);
   };
   R_Date("UGAMS-26433, R-12-7, ash layer",1150,25);
   Boundary("End Muru Ut Pata");
  };
 };
```

Ch'iji Jawira

```
 Plot()
 {
  Curve("IntCal20","IntCal20.14c");
  Curve("SHCal20","SHCal20.14c");
  Mix_Curves("Tiwanaku
mix","SHCal20","IntCal20",P(-1,101,[0,1,1,1,1,1,1,1,1,1,1,0,0,0,0,0,0,0,0,0,0,8,8,8,8,8,8,8
,8,8,8,20,20,20,20,20,20,20,20,20,20,20,20,20,20,20,20,20,20,20,20,11,11,11,11,11,11,1
1,11,11,11,9,9,9,9,9,9,9,9,9,9,2,2,2,2,2,2,2,2,2,2,0,0,0,0,0,0,0,0,0,0,0,0,0,0,0,0,0,0,0,0,0
]));
  Sequence("Individual CJ-35250")
  {
   Boundary("Birth CJ-35250");
   Interval("til eruption of Molar 1",U(5.5,6.5));
   R_Date("Poz-122666",1301,28);
   Interval("til eruption of Canine", U(2.7,7));
   R_Date("Poz-122665",1297,30);
   Interval("til Death",U(17.5,29.8));
   Boundary("Death CJ-35250");
  };
 };
```

Mollo Konu

```
Plot()
 {
  Curve("IntCal20","IntCal20.14c");
  Curve("SHCal20","SHCal20.14c");
  Mix_Curves("Tiwanaku
mix","SHCal20","IntCal20",P(-1,101,[0,1,1,1,1,1,1,1,1,1,1,0,0,0,0,0,0,0,0,0,0,8,8,8,8,8,8,8
,8,8,8,20,20,20,20,20,20,20,20,20,20,20,20,20,20,20,20,20,20,20,20,11,11,11,11,11,11,1
1,11,11,11,9,9,9,9,9,9,9,9,9,9,2,2,2,2,2,2,2,2,2,2,0,0,0,0,0,0,0,0,0,0,0,0,0,0,0,0,0,0,0,0,0
]));
  Sequence("Sector A")
  {
   Boundary("Start sector A");
   R_Date("Beta-275873, A91, III (surface A2)", 1350, 40);
   R_Date("Beta-275874, A34, II (surface A3)", 1330, 40);
   Phase("Surface A21")
   {
    R_Date("Beta-275871, A96, I (surface A21)", 1310, 40);
    R_Date("Beta-275872, A103, I (surface A21)", 1300, 40);
```

```
 };
 Boundary("End sector A");
};
//Sector A, no stratigraphic relationship
R_Date("Beta-275875, A111, I (pit midden, surface A21)", 1030, 40);
R_Combine("TW110")
{
 R_Date("Poz-122713",1117,29);
 R_Date("Poz-122714",1088,29);
};
Sequence("Sector C")
{
 Boundary("Start sector C");
 Phase("Sector C, 2001 excavations, Unit 5")
 {
  R_Combine("Feature 12, 2001 Excavations")
  {
   R_Date("AA-107592",1553,25);
   R_Date("AA-107593",1509,25);
  };
  R_Date("AA-107594, F19",1507,25);
  R_Date("AA-107595, F22",1508,25);
  First("First sector C");
  Last("Last sector C");
 };
 Boundary("End sector C");
};
Sequence("Sector D")
{
 Boundary("Start Sector D");
 R_Date("Beta-275868, D148, V (wall fill)", 1380, 40);
 R_Date("Beta-275869, D149, V (pit that cuts wall)", 1410, 40);
 Boundary("Boundary between occupations IV and IV");
 KDE_Plot("Occupation IV")
 {
  Sequence("Occupation III–IV")
  {
   R_Date("Beta-275867, D143, IV", 1110, 40);
   R_Date("Beta-275865, D139, IV", 1030, 40);
  };
  R_Date("Beta-275866, D156, IV", 1170, 40);
  R_Date("Beta-275863, D40, III", 1090, 40);
  R_Date("Beta-275864, D44, III", 1120, 40);
 };
 Date("End permanent residence");
 R_Date("Beta-275870, D36, I/II (latest)", 1070, 40);
```

```
  Boundary("End Sector D");
 };
 //Sector M (Mound) burials, no stratigraphic relationships
 R_Date("AA-107596, Burial M7",1150,25);
 R_Date("AA-107597, Burial M6",1006,26);
 R_Date("AA-107598, Burial M16",815,24);
 R_Date("AA-107599, Burial M8",706,24);
 R_Date("AA-107601, F10, 1991 excavations",1080,25);
 R_Date("AA-107602, F19, 1991 excavations",1089,25);
};
```

The Putuni complex

```
Plot()
{
 Curve("IntCal20","IntCal20.14c");
 Curve("SHCal20","SHCal20.14c");
 Mix_Curves("Tiwanaku
mix","SHCal20","IntCal20",P(-1,101,[0,1,1,1,1,1,1,1,1,1,1,0,0,0,0,0,0,0,0,0,0,8,8,8,8,8,8,8
,8,8,8,20,20,20,20,20,20,20,20,20,20,20,20,20,20,20,20,20,20,20,20,11,11,11,11,11,11,1
1,11,11,11,9,9,9,9,9,9,9,9,9,9,2,2,2,2,2,2,2,2,2,2,0,0,0,0,0,0,0,0,0,0,0,0,0,0,0,0,0,0,0,0,0
]));
 Sequence("Putuni")
 {
  Boundary("Start Putuni");
  R_Date("sub-Putuni, OS-11306",1560,25);
  KDE_Plot("Tiwanaku period occupation")
  {
   First("First Putuni");
   Last("Last Putuni");
   Sequence("Tiwanaku period occupation")
   {
    Phase("Earlier Tiwanaku occupation, below the main Putuni Platform")
    {
     R_Date("SMU-2369",1360,155);
     R_Date("Wk-49175, human bone in platform fill",1322,21);
     R_Date("AA-107591, giant urn burial",1352,25);
     R_Date("AA-107590, woman with elaborate grave goods",1337,25);
     R_Date("OS-10643",1230,40);
    };
    Combine("Feature 18, llamas fetus offering, Putuni")
    {
     R_Date("SMU-2467",1132,61);
     R_Date("SMU-2465",1105,53);
     R_Date("SMU-2466",1174,59);
```

```
   };
   R_Date("SMU-2472, collapsed roof beam",1197,115);
  };
 };
 Boundary("End Putuni");
};
};
```

Site-wide model of permanent and temporary residence

```
Plot()
{
 Curve("IntCal20","IntCal20.14c");
 Curve("SHCal20","SHCal20.14c");
 Mix_Curves("Tiwanaku
mix","SHCal20","IntCal20",P(-1,101,[0,1,1,1,1,1,1,1,1,1,1,0,0,0,0,0,0,0,0,0,0,8,8,8,8,8,8,8
,8,8,8,20,20,20,20,20,20,20,20,20,20,20,20,20,20,20,20,20,20,20,20,11,11,11,11,11,11,1
1,11,11,11,9,9,9,9,9,9,9,9,9,9,2,2,2,2,2,2,2,2,2,2,0,0,0,0,0,0,0,0,0,0,0,0,0,0,0,0,0,0,0,0
]));
 Sequence()
 {
  Boundary("Start Residential Dates");
  R_Date("sub-Putuni OS-11306",1560,25);
  KDE_Plot("Early Tiwanaku")
  {
   First("First Early Tiwanaku");
   Last("Last Early Tiwanaku");
   //Akapana East
   R_Date("SMU-2333, AKE1",1716,276);
   R_Date("Beta-55491, AKE1 Mound",1500,100);
   R_Date("SMU-2471, AKE1 (not mound)",1428,99);
   //Mollo Kontu
   Sequence("Mollo Kontu")
   {
    R_Date("Beta-275868, D148, V (wall fill)", 1380, 40);
    R_Date("Beta-275869, D149, V (pit that cuts wall)", 1410, 40);
   };
   //Putuni
   R_Date("SMU-2369",1360,155);
   R_Date("Wk-49175, human bone in platform fill",1322,21);
   R_Date("AA-107591, giant urn burial",1352,25);
   R_Date("AA-107590, woman with elaborate grave goods",1337,25);
   R_Date("OS-10643, use surface, Putuni",1230,40);
  };
  KDE_Plot("Late Tiwanaku")
  {
```

```
  First("First Late Tiwanaku");
  Last("Last Late Tiwanaku");
  //Akapana East
  R_Date("SMU-2469, AKE1",1191,101);
  R_Date("AKE1, SMU-2277, AKE1",1134,60);
  R_Date("SMU-2278, AKE1",1246,112);
  R_Date("SMU-2289, AKE1",1185,56);
  R_Date("SMU-2276, AKE1",1073,57);
  R_Date("SMU-2290, AKE1",1121,74);
  //Muru Ut Pata
  Sequence("Muru Ut Pata")
  {
   Phase()
   {
    R_Date("UGAMS-26434, R-13-8, principal occupation",1110,20);
    R_Date("UGAMS-26432, R-10-14, shaft tomb, individual 11",1120,20);
   };
   R_Date("UGAMS-26433, R-12-7, ash layer",1150,25);
  };
  //Mollo Kontu
  Sequence("Mollo Kontu Occupation III–IV")
  {
   R_Date("Beta-275867, D143, IV", 1110, 40);
   R_Date("Beta-275865, D139, IV", 1030, 40);
  };
  R_Date("Beta-275866, D156, IV", 1170, 40);
  R_Date("Beta-275863, D40, III", 1090, 40);
  R_Date("Beta-275864, D44, III", 1120, 40);
  Sequence("Putuni")
  {
   Combine("Putuni F18")
   {
    R_Date("SMU-2467",1132,61);
    R_Date("SMU-2465",1105,53);
    R_Date("SMU-2466",1174,59);
   };
   R_Date("SMU-2472, roof beam",1197,115);
  };
 };
 Boundary("End permanent residence");
 KDE_Plot("Terminal Tiwanaku")
 {
  First("First Terminal Tiwanaku");
  Last("Last Terminal Tiwanaku");
  Combine("Manzanilla House")
  {
```

```
    R_Date("SMU-2473, F11. Offering above square structure", 850, 243);
    R_Date("INAH-972, F11",1120,140);
   };
   //Akapana East 1 Mound
   R_Date("SMU-2332, AKE1",1017,115);
   R_Date("SMU-2331, AKE1",872,197);
   R_Date("SMU-2740 AKE1",632,183);
   //Mollo Kontu
   R_Date("Beta-275870, D36, I/II (latest)", 1070, 40);
   R_Date("Beta-275875, A111, I (pit midden, surface A21)", 1030, 40);
  };
  Boundary("End");
 };
};
```

Pumapunku

```
Plot()
{
 Curve("IntCal20","IntCal20.14c");
 Curve("SHCal20","SHCal20.14c");
 Mix_Curves("Tiwanaku
mix","SHCal20","IntCal20",P(-1,101,[0,1,1,1,1,1,1,1,1,1,1,0,0,0,0,0,0,0,0,0,0,8,8,8,8,8,8,8
,8,8,8,20,20,20,20,20,20,20,20,20,20,20,20,20,20,20,20,20,20,20,20,11,11,11,11,11,11,1
1,11,11,11,9,9,9,9,9,9,9,9,9,9,2,2,2,2,2,2,2,2,2,2,0,0,0,0,0,0,0,0,0,0,0,0,0,0,0,0,0,0,0,0,0
]));
 Sequence("Pumapunku")
 {
  Boundary("Begin construction");
  KDE_Plot("Group 1")
  {
   First("Group 1 First");
   Last("Group 1 Last");
   Sequence()
   {
    Phase("Foundation")
    {
     R_Date("OS-17860",1510,25);
     R_Date("AA-65280",1424,83);
    };
    Date("Green surface");
    Phase("Between green and red surface")
    {
     R_Date("UCIAMS-62884, P-11",1445,20);
     R_Date("UCIAMS-62885",1370,20);
    };
```

```
  Date("Red surface");
  Phase("P-8, above red surface")
  {
   R_Date("UCIAMS-62886",1315,30);
   R_Combine("TW059, petrous bone, Northwest sector 1, Locus 2. Platform fill")
   {
    R_Date("Poz-123574", 1267,28);
    R_Date("Poz-123630", 1300,29);
   };
  };
 };
};
KDE_Plot("Group 2 - looting events")
{
 R_Date("AA-65282",1284,40);
 R_Date("AA-68185",1251,36);
 R_Date("AA-68183",1241,37);
};
Boundary("End Tiwanaku period use");
Boundary("Start Post-construction disturbance");
KDE_Plot("Post-construction disturbance")
{
 R_Combine("TW100")
 {
  R_Date("Poz-123667", 1028,30);
  R_Date("Poz-123668", 953,30);
 };
 R_Date("AA-68184, South Side, unit M-3, feature 320, looter's pit",1046,33);
};
Boundary("End Post-construction disturbance");
Boundary("Start Inca occupation");
KDE_Plot("Inca")
{
 R_Date("AA-68178",315,33);
 R_Date("AA-65283",322,54);
 R_Date("AA-68182",356,36);
 R_Date("AA-68179",362,35);
 R_Date("AA-68180",365,31);
 R_Date("AA-68181",350,31);
 R_Date("AA-65286",393,35);
 First("Inca First");
 Last("Inca Last");
 Span("Inca Span");
};
KDE_Plot("Early Colonial, unit C-29 feature 246")
{
```

```
   R_Date("AA-65278",322,55);
   R_Date("AA-65279",302,40);
   First("Colonial First");
   Last("Colonial Last");
   Span("Colonial Span");
  };
  Boundary("End Colonial occupation");
 };
};
```

Monument Construction

```
 Plot()
 {
  Curve("IntCal20","IntCal20.14c");
  Curve("SHCal20","SHCal20.14c");
  Mix_Curves("Tiwanaku
mix","SHCal20","IntCal20",P(-1,101,[0,1,1,1,1,1,1,1,1,1,1,0,0,0,0,0,0,0,0,0,0,8,8,8,8,8,8,8
,8,8,8,20,20,20,20,20,20,20,20,20,20,20,20,20,20,20,20,20,20,20,20,11,11,11,11,11,11,1
1,11,11,11,9,9,9,9,9,9,9,9,9,9,2,2,2,2,2,2,2,2,2,2,0,0,0,0,0,0,0,0,0,0,0,0,0,0,0,0,0,0,0,0,0
]));
  Sequence("Pumapunku & Akapana")
  {
   Boundary("Start");
   KDE_Plot("Monument construction")
   {
    //Akapana
    R_Date("SMU-2329, at base near canal mouth", 1403, 211);
    R_Date("SMU-2468, summit", 1392, 49);
    //Just below kero smash, level 6b
    R_Date("ETH-6306", 1460, 60);
    //fill in between facade and revetment on the first terrace
    Combine("Surface of terrace 1. Level 6a, F2. Wall 2. Ceramic smash")
    {
     R_Date("SMU-2285, on top of first platform", 1425, 211);
     R_Date("SMU-2293, on top of first platform", 1388, 116);
    };
    //Pumapunku
    Sequence()
    {
     Phase("Foundation")
     {
      R_Date("OS-17860",1510,25);
      R_Date("AA-65280",1424,83);
     };
```

```
    Date("Green surface");
    Phase("Between green and red surface")
    {
     R_Date("UCIAMS-62884, P-11",1445,20);
     R_Date("UCIAMS-62885",1370,20);
    };
    Date("Red surface");
    Phase("P-8, above red surface")
    {
     R_Date("UCIAMS-62886",1315,30);
     R_Combine("TW059, petrous bone, Northwest sector 1, Locus 2. Platform fill")
     {
      R_Date("Poz-123574", 1267,28);
      R_Date("Poz-123630", 1300,29);
     };
    };
   };
  };
  Boundary("End");
 };
 Sequence("Depositional sequence north of the Akapana")
 {
  Boundary();
  R_Date("TW097, Wk-50229, body within pebble surface", 1184, 14);
  Date("Pebble surface placed");
  R_Combine("TW090, Verano, Ind. 6")
  {
   R_Date("Poz-122663",1039,30);
   R_Date("Poz-122664",1054,28);
  };
  Boundary();
 };
 Sequence()
 {
  Boundary();
  KDE_Plot("Late Tiwanaku community building")
  {
   Date("=Pebble surface placed");
   Sequence()
   {
    Combine("Feature 18, llamas fetus offering, Putuni")
    {
     R_Date("SMU-2467",1132,61);
     R_Date("SMU-2465",1105,53);
     R_Date("SMU-2466",1174,59);
    };
```

```
    //Couture 2002:262
    R_Date("SMU-2472, collapsed roof beam",1197,115);
   };
  };
  Boundary();
 };
};
```

Akapana

```
 Plot()
 {
  Curve("IntCal20","IntCal20.14c");
  Curve("SHCal20","SHCal20.14c");
  Mix_Curves("Tiwanaku
mix","SHCal20","IntCal20",P(-1,101,[0,1,1,1,1,1,1,1,1,1,1,0,0,0,0,0,0,0,0,0,0,8,8,8,8,8,8,8
,8,8,8,20,20,20,20,20,20,20,20,20,20,20,20,20,20,20,20,20,20,20,20,11,11,11,11,11,11,1
1,11,11,11,9,9,9,9,9,9,9,9,9,9,2,2,2,2,2,2,2,2,2,2,0,0,0,0,0,0,0,0,0,0,0,0,0,0,0,0,0,0,0,0,0
]));
  Sequence()
  {
   Boundary("Start construction and early offerings");
   KDE_Plot("Construction and early offerings")
   {
    R_Date("SMU-2329, at base near canal mouth", 1403, 211);
    R_Date("SMU-2468, summit", 1392, 49);
    R_Date("ETH-6306, level 6b", 1460, 60);
    Combine("Surface of terrace 1. Level 6a, F2. Wall 2. Ceramic smash")
    {
     R_Date("SMU-2285, on top of first platform", 1425, 211);
     R_Date("SMU-2293, on top of first platform", 1388, 116);
    };
   };
   Boundary("End Construction and early offerings");
  };
  //Summit
  R_Date("SMU-2336, sala sur",1243,113);
  R_Date("AA-107584, burial with puma head ceramics",1259,26);
  Combine("Manzanilla House")
  {
   R_Date("SMU-2473, F11. Offering above square structure", 850, 243);
   R_Date("INAH-972, F11",1120,140);
  };
```

```
 //Some dates not from the Akapana; included here so they can be combined and/or
calibrated before exporting the posteriors
 //Northwest corner, dates from the 1990s
 R_Date("SMU-2367, terrace 1 offering ", 1152, 78);
 R_Date("SMU-2330, base, carnivore at canal mouth", 1079, 109);
 R_Date("ETH-5640, terrace 2, offering", 1170, 65);
 Sequence("Ceramic smash and overlying bones")
 {
  Date("=Surface of terrace 1. Level 6a, F2. Wall 2. Ceramic smash");
  R_Date("ETH-5639, bones above ceramic smash", 1170, 60);
 };
 //Northwest corner, recently processed dates
 R_Combine("TW001, Feature 6225")
 {
  R_Date("Poz-122654", 1060,30);
  R_Date("Poz-122655", 1141,30);
 };
 R_Date("TW006, Poz-122657, base of terrace 1", 1142, 22);
 R_Date("TW060, Wk-49174", 1109, 20);
 R_Combine("TW061, Feature 6225")
 {
  R_Date("Poz-123576", 993,27);
  R_Date("Poz-123631", 1012,26);
 };
 R_Combine("TW065, 16.A.25, F55")
 {
  R_Date("Poz-122660",1124,30);
  R_Date("Poz-122661",1149,29);
 };
 R_Combine("TW102, locus 643. Disturbed context")
 {
  R_Date("Poz-122711",1024,29);
  R_Date("Poz-122712",991,38);
 };
 R_Date("Ind. I0978, OxA-31443", 969,28);
 R_Date("Ind. I0977, OxA-31463", 1056,23);
 //Group burial northeast of the Akapana
 Sequence("Verano")
 {
  R_Date("TW097, Wk-50229, body within pebble surface", 1184, 14);
  Date("Pebble surface placed");
  R_Combine("TW090, Verano, Ind 6")
  {
   R_Date("Poz-122663",1039,30);
   R_Date("Poz-122664",1054,28);
  };
```

```
 };
 //Monolito descabezado / Headless monolith
 R_Date("TW056, Ind 3, Wk-49173", 1158, 20);
 R_Combine("TW064, Ind 4")
 {
  R_Date("Poz-122658",1100,29);
  R_Date("Poz-122659",1163,30);
 };
 //No contextual information
 R_Date("TW004, Wk-49169, Akapana PAAK 2004 (northwest corner?)", 1186, 22);
 R_Date("TW008, Wk-49170, excavation for museum", 1183, 21);
};
```

Redwares

```
};
Plot()
{
 //Priors are imported from other models and already calibrated
 Sequence()
 {
  Boundary("Start Redwares");
  KDE_Plot("Redwares")
  {
   First("First Redwares");
   Last("Last Redwares");
   Span("Span Redwares");
   Date(Prior("ETH_6306","ETH_6306.prior"));
   Date(Prior("Surface_of_terrace_1","Surface_of_terrace_1.prior"));
Date(Prior("SMU_2329_at_base_near_canal_mouth","SMU_2329_at_base_near_canal_mou
th.prior"));
   Date(Prior("SMU_2336_sala_sur","SMU_2336_sala_sur.prior"));
   Date(Prior("AA_107584_puma_head_burial","AA_107584_puma_head_burial.prior"));
Date(Prior("ETH_5639_bones_above_kero_smash","ETH_5639_bones_above_kero_smash.p
rior"));
   Date(Prior("ETH_5640_terrace_2_offering","ETH_5640_terrace_2_offering.prior"));
   Date(Prior("Manzanilla_House","Manzanilla_House.prior"));
   Date(Prior("TW060_Wk_49174","TW060_Wk_49174.prior"));
   Date(Prior("TW090_Verano_Ind","TW090_Verano_Ind.prior"));
   Date(Prior("TW056_Ind_3_Wk_49173","TW056_Ind_3_Wk_49173.prior"));

Date(Prior("TW064_Ind_4_Monolito_descabezado","TW064_Ind_4_Monolito_descabezado.p
rior"));
```

```
Date(Prior("Beta_275871_A96_I_surface_A21_","Beta_275871_A96_I_surface_A21_.prior")
);
    Date(Prior("Beta_275875_A111","Beta_275875_A111.prior"));
    Date(Prior("Beta_275863_D40_III","Beta_275863_D40_III.prior"));
    Date(Prior("Beta_275864_D44_III","Beta_275864_D44_III.prior"));
    Date(Prior("Beta_275865_D139_IV","Beta_275865_D139_IV.prior"));
    Date(Prior("Beta_275866_D156_IV","Beta_275866_D156_IV.prior"));
    Date(Prior("Beta_275867_D143_IV","Beta_275867_D143_IV.prior"));
    Date(Prior("Beta_275868_D148_V_wall_fill_","Beta_275868_D148_V_wall_fill_.prior"));

Date(Prior("Beta_275869_D149_V_pit_that_cuts_wall_","Beta_275869_D149_V_pit_that_c
uts_wall_.prior"));
    Date(Prior("Beta_275870_D36","Beta_275870_D36.prior"));
    Date(Prior("Beta_275875_A111","Beta_275875_A111.prior"));
    Date(Prior("TW110","TW110.prior"));
    Date(Prior("AA-65280","AA_65280.prior"));

Date(Prior("AA_107586_three_chambered_burial","AA_107586_three_chambered_burial.pri
or"));
    Date(Prior("SMU_2471_AKE1_not_mound_","SMU_2471_AKE1_not_mound_.prior"));
    Date(Prior("SMU_2278_AKE1","SMU_2278_AKE1.prior"));
    Date(Prior("SMU_2469_AKE1","SMU_2469_AKE1.prior"));
    Date(Prior("SMU_2289_AKE1","SMU_2289_AKE1.prior"));
    Date(Prior("AKE1_SMU_2277_AKE1","AKE1_SMU_2277_AKE1.prior"));
    Date(Prior("SMU_2290_AKE1","SMU_2290_AKE1.prior"));
    Date(Prior("SMU_2276_AKE1","SMU_2276_AKE1.prior"));
    Date(Prior("SMU_2740_AKE1","SMU_2740_AKE1.prior"));
    Date(Prior("Beta_55491_AKE1_Mound","Beta_55491_AKE1_Mound.prior"));

Date(Prior("UGAMS_26434_R_13_8_principal_occupation","UGAMS_26434_R_13_8_princip
al_occupation.prior"));

Date(Prior("UGAMS_26432_R_10_14_shaft_tomb_individual_11","UGAMS_26432_R_10_14
_shaft_tomb_individual_11.prior"));

Date(Prior("UGAMS_26433_R_12_7_ash_layer","UGAMS_26433_R_12_7_ash_layer.prior"))
;

Date(Prior("Wk_49175_human_bone_in_platform_fill","Wk_49175_human_bone_in_platfor
m_fill.prior"));

Date(Prior("AA_107590_woman_with_elaborate_grave_goods","AA_107590_woman_with_e
laborate_grave_goods.prior"));
    Date(Prior("AA_107591_giant_urn_burial","AA_107591_giant_urn_burial.prior"));
    Date(Prior("SMU_2369","SMU_2369.prior"));
```

```
    Date(Prior("OS_10643","OS_10643.prior"));

Date(Prior("SMU_2472_collapsed_roof_beam","SMU_2472_collapsed_roof_beam.prior"));

Date(Prior("F18_llamas_fetus_offering_Putuni","F18_llamas_fetus_offering_Putuni.prior"));
   };
   Boundary("End Redwares");
  };
 };
```

All contexts with human bone

```
 Plot()
 {
  //Priors are imported from other models and already calibrated
  Sequence()
  {
   Boundary("Start human bone");
   KDE_Plot("Contexts with human bone")
   {
    First("First human bone");
    Last("Last human bone");
    Date(Prior("ETH_6306","ETH_6306.prior"));

Date(Prior("SMU_2329_at_base_near_canal_mouth","SMU_2329_at_base_near_canal_mou
th.prior"));
    Date(Prior("SMU_2468_summit","SMU_2468_summit.prior"));

Date(Prior("ETH_5639_bones_above_kero_smash","ETH_5639_bones_above_kero_smash.p
rior"));
    Date(Prior("ETH_5640_terrace_2_offering","ETH_5640_terrace_2_offering.prior"));

Date(Prior("TW008_Wk_49170_excavation_for_museum","TW008_Wk_49170_excavation_f
or_museum.prior"));

Date(Prior("TW004_Wk_49169_Akapana_PAAK_2004_northwest_corner_","TW004_Wk_491
69_Akapana_PAAK_2004_northwest_corner_.prior"));
    Date(Prior("AA_107584_puma_head_burial","AA_107584_puma_head_burial.prior"));
    Date(Prior("TW102_locus_643","TW102_locus_643.prior"));
    Date(Prior("Ind_I0977_OxA_31463","Ind_I0977_OxA_31463.prior"));
    Date(Prior("Ind_I0978_OxA_31443","Ind_I0978_OxA_31443.prior"));
    Date(Prior("TW006_base_of_terrace_1","TW006_base_of_terrace_1.prior"));
    Date(Prior("TW065_16","TW065_16.prior"));
    Date(Prior("TW061_Feature_6225","TW061_Feature_6225.prior"));
    Date(Prior("TW090_Verano_Ind","TW090_Verano_Ind.prior"));
```

```
    Date(Prior("TW056_Ind_3_Wk_49173","TW056_Ind_3_Wk_49173.prior"));

Date(Prior("TW097_Wk_50229_body_within_pebble_surface","TW097_Wk_50229_body_wit
hin_pebble_surface.prior"));
    Date(Prior("TW059_Ind","TW059_Ind.prior"));

Date(Prior("TW064_Ind_4_Monolito_descabezado","TW064_Ind_4_Monolito_descabezado.p
rior"));
    Date(Prior("CJ_35250_TW099","CJ_35250_TW099.prior"));
    Date(Prior("Feature_12_2001_Excavations","Feature_12_2001_Excavations.prior"));
    Date(Prior("AA_107594_F19","AA_107594_F19.prior"));
    Date(Prior("AA_107595_F22","AA_107595_F22.prior"));
    Date(Prior("AA_107596_Burial_M7","AA_107596_Burial_M7.prior"));
    Date(Prior("AA_107597_Burial_M6","AA_107597_Burial_M6.prior"));
    Date(Prior("AA_107598_Burial_M16","AA_107598_Burial_M16.prior"));
    Date(Prior("AA_107599_Burial_M8","AA_107599_Burial_M8.prior"));

Date(Prior("Beta_275873_A91_III_surface_A2_","Beta_275873_A91_III_surface_A2_.prior"
));

Date(Prior("AA_107601_F10_1991_excavations","AA_107601_F10_1991_excavations.prior"
));

Date(Prior("AA_107602_F19_1991_excavations","AA_107602_F19_1991_excavations.prior"
));

Date(Prior("Beta_275871_A96_I_surface_A21_","Beta_275871_A96_I_surface_A21_.prior")
);

Date(Prior("Beta_275872_A103_I_surface_A21_","Beta_275872_A103_I_surface_A21_.prio
r"));

Date(Prior("Beta_275874_A34_II_surface_A3_","Beta_275874_A34_II_surface_A3_.prior"))
;
    Date(Prior("TW110","TW110.prior"));
    Date(Prior("TW100_Pumapunku_burial","TW100_Pumapunku_burial.prior"));
    Date(Prior("TW059_Ind","TW059_Ind.prior"));
    Date(Prior("AA_107585_cyst_burial","AA_107585_cyst_burial.prior"));

Date(Prior("AA_107586_three_chambered_burial","AA_107586_three_chambered_burial.pri
or"));
    Date(Prior("SMU_2333_AKE1","SMU_2333_AKE1.prior"));

Date(Prior("Wk_49175_human_bone_in_platform_fill","Wk_49175_human_bone_in_platfor
m_fill.prior"));
```

```
Date(Prior("AA_107590_woman_with_elaborate_grave_goods","AA_107590_woman_with_e
laborate_grave_goods.prior"));
    Date(Prior("AA_107591_giant_urn_burial","AA_107591_giant_urn_burial.prior"));

Date(Prior("F18_llamas_fetus_offering_Putuni","F18_llamas_fetus_offering_Putuni.prior"));
   };
   Boundary("End human bone");
  };
 };
```

Tombs, Violent deaths, and other contexts with human bone

```
 Plot()
 {
  //Priors are imported from other models and already calibrated
  Sequence()
  {
   Boundary("Start Tombs");
   KDE_Plot("Tombs")
   {
    First("First Tombs");
    Last("Last Tombs");
    Span("Span Tombs");
    Date(Prior("AA_107584_puma_head_burial","AA_107584_puma_head_burial.prior"));
    Date(Prior("CJ_35250_TW099","CJ_35250_TW099.prior"));
    Date(Prior("Feature_12_2001_Excavations","Feature_12_2001_Excavations.prior"));
    Date(Prior("AA_107594_F19","AA_107594_F19.prior"));
    Date(Prior("AA_107595_F22","AA_107595_F22.prior"));

Date(Prior("Beta_275871_A96_I_surface_A21_","Beta_275871_A96_I_surface_A21_.prior")
);

Date(Prior("Beta_275872_A103_I_surface_A21_","Beta_275872_A103_I_surface_A21_.prio
r"));
    Date(Prior("AA_107585_cyst_burial","AA_107585_cyst_burial.prior"));

Date(Prior("AA_107586_three_chambered_burial","AA_107586_three_chambered_burial.pri
or"));

Date(Prior("AA_107590_woman_with_elaborate_grave_goods","AA_107590_woman_with_e
laborate_grave_goods.prior"));
    Date(Prior("AA_107591_giant_urn_burial","AA_107591_giant_urn_burial.prior"));

Date(Prior("F18_llamas_fetus_offering_Putuni","F18_llamas_fetus_offering_Putuni.prior"));
   };
```

```
  Boundary("End Tombs");
 };
 Sequence()
 {
  Boundary("Start Violent Deaths");
  KDE_Plot("Violent Deaths")
  {
   First("First Violent Deaths");
   Last("Last Violent Deaths");
   Span("Span Violent Deaths");
   Date(Prior("TW056_Ind_3_Wk_49173","TW056_Ind_3_Wk_49173.prior"));

Date(Prior("TW064_Ind_4_Monolito_descabezado","TW064_Ind_4_Monolito_descabezado.p
rior"));

Date(Prior("AA_107602_F19_1991_excavations","AA_107602_F19_1991_excavations.prior"
));

Date(Prior("ETH_5639_bones_above_kero_smash","ETH_5639_bones_above_kero_smash.p
rior"));
   Date(Prior("ETH_5640_terrace_2_offering","ETH_5640_terrace_2_offering.prior"));
   Date(Prior("TW090_Verano_Ind","TW090_Verano_Ind.prior"));

Date(Prior("TW097_Wk_50229_body_within_pebble_surface","TW097_Wk_50229_body_wit
hin_pebble_surface.prior"));
  };
  Boundary("End Violent Deaths");
 };
 Sequence()
 {
  Boundary("Start human bone, NOT tomb or violent deaths");
  KDE_Plot("Human bone, NOT tomb or violent deaths")
  {
   First("First bone NOT tomb or violent death");
   Last("Last bone NOT tomb or violent death");

Date(Prior("AA_107601_F10_1991_excavations","AA_107601_F10_1991_excavations.prior"
));
   Date(Prior("TW110","TW110.prior"));
   Date(Prior("ETH_6306","ETH_6306.prior"));

Date(Prior("SMU_2329_at_base_near_canal_mouth","SMU_2329_at_base_near_canal_mou
th.prior"));
   Date(Prior("SMU_2468_summit","SMU_2468_summit.prior"));
```

```
Date(Prior("TW008_Wk_49170_excavation_for_museum","TW008_Wk_49170_excavation_f
or_museum.prior"));

Date(Prior("TW004_Wk_49169_Akapana_PAAK_2004_northwest_corner_","TW004_Wk_491
69_Akapana_PAAK_2004_northwest_corner_.prior"));
    Date(Prior("TW102_locus_643","TW102_locus_643.prior"));
    Date(Prior("Ind_I0977_OxA_31463","Ind_I0977_OxA_31463.prior"));
    Date(Prior("Ind_I0978_OxA_31443","Ind_I0978_OxA_31443.prior"));
    Date(Prior("TW006_base_of_terrace_1","TW006_base_of_terrace_1.prior"));
    Date(Prior("TW006_base_of_terrace_1","TW006_base_of_terrace_1.prior"));
    Date(Prior("TW065_16","TW065_16.prior"));
    Date(Prior("TW061_Feature_6225","TW061_Feature_6225.prior"));
    Date(Prior("TW059_Ind","TW059_Ind.prior"));
    Date(Prior("AA_107596_Burial_M7","AA_107596_Burial_M7.prior"));
    Date(Prior("AA_107597_Burial_M6","AA_107597_Burial_M6.prior"));
    Date(Prior("AA_107598_Burial_M16","AA_107598_Burial_M16.prior"));
    Date(Prior("AA_107599_Burial_M8","AA_107599_Burial_M8.prior"));

Date(Prior("Beta_275873_A91_III_surface_A2_","Beta_275873_A91_III_surface_A2_.prior"
));
    Date(Prior("TW100_Pumapunku_burial","TW100_Pumapunku_burial.prior"));
    Date(Prior("SMU_2333_AKE1","SMU_2333_AKE1.prior"));

Date(Prior("Wk_49175_human_bone_in_platform_fill","Wk_49175_human_bone_in_platfor
m_fill.prior"));
   };
   Boundary("End Human bone, NOT tomb or violent deaths");
  };
 };
```
