# Supplementary material for: The center cannot hold: A Bayesian chronology for the collapse of Tiwanaku
Source: PLoS One. 2023 Nov 22;18(11):e0288798. doi: 10.1371/journal.pone.0288798 (PMC10664893; doi:10.1371/journal.pone.0288798)
Supplement: S5 File — Available from https://osf.io/v6j7n/. (PDF) [file pone.0288798.s006.pdf]

Supplementary file for:

Marsh EJ, Vranich A, Blom D, Bruno M, Davis K, Augustine J, et al. The center cannot hold: A Bayesian chronology for the collapse of Tiwanaku. PLOS ONE. 2023;18: e0288798. doi:10.1371/journal.pone.0288798

S5. Orthomosaic of 1930s aerial photographs

The aerial imagery used in this paper is a composite orthomosaic stored at Open Science Foundations: https://osf.io/v6j7n/. It was made with scans from three sources, which all seem to be incomplete sets of aerial photographs that Wendell Bennett probably commissioned during one of his two expeditions to Bolivia in 1932 and 1933–1934, but this has not been confirmed. We think that the most likely date is the first half of 1934, when Junius Bird accompanied Bennett as a photographer for the first five months. They may have been inspired by the success of the 1931 Shippee–Johnson expedition to photograph archaeological sites in Peru [1, 2]. The Shippee–Johnson photographs are curated by the American Museum of Natural History, where Bennett worked at the time. This museum may have additional photographs, films, or documents. These are the three sources of images we worked with:

1. In 2013, John Janusek scanned prints of photographs that Charles Ortloff had obtained from Alan Sawyer's private collection. These scans were provided courtesy of Anna Guengerich.
2. We used scans from the negatives from the Sawyer collection, now held at the University of British Columbia in Vancouver, courtesy of Erwin Wodarczak and Candice Bjur in the University Archives section. The images are from item 16-26 (this item includes five images taken from other archaeological sites, perhaps Chan Chan and the Nasca region).
   Holdings details: https://webcat.library.ubc.ca/vwebv/holdingsInfo?bibId=7894884
   Sawyer collection inventory: https://www.library.ubc.ca/archives/u_arch/sawyer.pdf
3. We also used scans of the prints that Sawyer made copies of in 1959, courtesy of the Division of Anthropology, Peabody Museum of Natural History, Yale University. These are part of the extensive Bennett archive, which has not been fully processed. Some images were found with index cards dated to 1932.

In 1955, Alan Sawyer excavated at Tiwanaku with his graduate advisor, Alfred Kidder II, who submitted the site's first radiocarbon dates [3]. In 1959, Sawyer obtained copies of these images from the Peabody Museum of Natural History, according to documentation in the Sawyer collection (he did not commission the images, as suggested by [4]). Later, Charless Ortloff made copies from Sawyer's collection, which Janusek scanned in 2013. A mosaic of these images appears in a few publications,

which Janusek manually assembled in Photoshop with no image corrections [5, 6]. These images are called the Sawyer photographs without further information about their source. They are used more extensively by Janusek and Bowen [4]. They are some of the only images that show Tiwanaku prior to questionable reconstruction efforts beginning in the 1960s [7]. The water table was higher at the time, which means water features are more apparent [4].

For this publication, we combined all three sets of images into a composite orthomosaic. We chose 87 images from our three sources, removing low-quality and repeated photographs from the original set of 98. We cropped the photographs to remove edges and handwritten numbers. In Metashape 1.8, we oriented the photographs at maximum quality and optimized the cameras before creating a mesh using 2.5D relief with a single face. We smoothed and decimated the mesh with a strength of 100 to reduce distortion. In creating the orthomosaic, we used the following options: refine seamlines, hole filling, ghosting filter, and back-face culling. This final orthomosaic retains the original image quality, but there are some minor distortions and image artifacts. There is more distortion near the edges of the orthomosaic, where there is less overlap in the original photographs. Finally, we manually georeferenced this image in QGIS 3.2 with 35 reference points, based on landmarks that are visible in more recent aerial and satellite photographs.

Future work should also consider other sets of aerial images from 1954–1955, 1972, 1989, and 1992 [8]. In the 2000s, Alexei Vranich coordinated efforts to produce two orthoimages and DEMs, based on photographs taken in 1972 and 1992 [9]. These data are made available by the Center for Advanced Spatial Technologies, University of Arkansas, but are not georeferenced.

1972 orthophoto:
https://gmv.cast.uark.edu/scanning/tiwanaku-bolivia-photogrammetry-of-area-in-1972-2/
1972 DEM based on the orthophoto. We use this DEM as a hillshaded layer in Fig 1, which includes shading for the reconstructed Kalasasaya, even though the overlaying image pre-dates the reconstruction.
https://gmv.cast.uark.edu/scanning/tiwanaku-boliviadem-2/
1992 orthophoto:
https://gmv.cast.uark.edu/scanning/tiwanaku-bolivia-photogrammetry-of-area-in-1992-2/
1992 DEM based on the orthophoto:
https://gmv.cast.uark.edu/scanning/tiwanaku-bolivia-digital-elevation-model-1992-2/

Finally, Johann Reinhard took ten images in 1989 that have not been processed. Some have appeared in print [10–12]. His personal website includes links to these photographs.

Photo archives from Bolivia:
https://www.johanreinhard.net/photos-videos/photo-archives#h.60r5tv7th48b
Photographs from Tiwanaku:
https://photos.google.com/share/AF1QipMp4SQj5CmfNWq_m8w5k_2XVB7Y0Ps8ViUnxBQS_lvRcmvvWvKuDpVchIwb6sFnKw?key=ekx3SE1EUVI3MHJuakFoT0pEaVZNeXNEdm5EaWlR

References

1. Denevan WM. The 1931 Shippee–Johnson Aerial Photography Expedition to Peru. Geographical Review. 1993;83: 238–251. doi:10.2307/215727
2. Weens J. "Wings over the Andes": Aerial Photography and the Dematerialization of Archaeology circa 1931. In: Pillsbury J, editor. Past presented: archaeological illustration and the ancient Americas. Washington, D.C.: Dumbarton Oaks; 2012. pp. 319–353.
3. Ralph EK. University of Pennsylvania Radiocarbon Dates III. American Journal of Science Radiocarbon Supplement. 1959;1: 45–58. doi:10.1017/S003382220002035X
4. Janusek J, Bowen C. Tiwanaku as Telluric Waterscape: Water and Stone in a Highland Andean City. In: Jennings J, Swenson E, editors. Powerful Places in the Ancient Andes. Albuquerque: University of New Mexico Press; 2018. pp. 209–246. Available: https://muse.jhu.edu/book/60581
5. Ortloff CR. Groundwater Management in the 300 bce-1100ce Pre-Columbian City of Tiwanaku (Bolivia). Hydrol Current Res. 2014;5: 2.
6. Ortloff CR, Janusek JW. Hydrologic Engineering of the Tiwanaku. In: Selin H, editor. Encyclopaedia of the History of Science, Technology, and Medicine in Non-Western Cultures. Dordrecht, The Netherlands: Springer; 2014. pp. 2267–2281. doi:10.1007/978-94-007-3934-5_10323-1
7. Protzen J-P, Nair S. The stones of Tiahuanaco: a study of architecture and construction. Los Angeles: Cotsen Institute of Archaeology Press, University of California, Los Angeles; 2013.
8. Marc-Antoine V, Nicolas L. Geomorphological map of the Tiwanaku River watershed in Bolivia: Implications for past and present human occupation. CATENA. 2021;206: 105508. doi:10.1016/j.catena.2021.105508
9. Cothren J, Goodmaster C, Barnes A, Ernenwein E, Vranich A, Limp W, et al. Fusion of Three-Dimensional Data at Tiwanaku: An Approach to Spatial Data Integration. In: Jerem E, Redő F, Szeverényi V, editors. On the Road to Reconstructing the Past: Computer Applications and Quantitative Methods in Archaeology (CAA). Budapest: Archaeolingua; 2011. Available: https://proceedings.caaconference.org/files/2008/00_Intro_CAA_2008.pdf
10. Blom DE, Janusek JW. Making Place: Humans as Dedications in Tiwanaku. World Archaeology. 2004;36: 123–141.

11. Reinhard J. Tiwanaku: Ensayo sobre su cosmovisión. Pumapunku (Nueva Época). 1991;1: 9–66.
12. Reinhard J. Tiahuanaco, Sacred Center of the Andes. In: McFarren P, editor. Lima: Fundación Quipus; 1990. pp. 151–181.
